# Toward sustainable plastic bioremediation using bacterial consortia from aquatic environments

**DOI:** 10.3389/fbioe.2025.1709072

**Published:** 2026-01-05

**Authors:** Maha Alharbi, Safaa Mohamed Abd-elhaliem, Salwa S. Afifi, walaa A. Al-shareef, Rasha A. Mosbah, Abdallah Tageldein Mansour, Nada K. Alharbi, Mahmoud M. Bendary

**Affiliations:** 1 Department of Biology, College of Science, Princess Nourah Bint Abdulrahman University, Riyadh, Saudi Arabia; 2 Microbiology and Immunology Department, Faculty of Pharmacy, Al-Azhar University, Nasr City, Egypt; 3 Microbiology and Immunology Department, Faculty of Pharmacy, October 6 University, 6th of October City, Egypt; 4 Infection Control Unit, Zagazig University Hospital, Zagazig, Egypt; 5 Fish and Animal Production Department, College of Agriculture and Food Sciences, King Faisal University, Al-Ahsa, Saudi Arabia; 6 Department of Microbiology and Immunology, Faculty of Pharmacy, Port Said University, Port Said, Egypt

**Keywords:** plastic, aquatic, biodegradation, bioremediation, eco-friendly

## Abstract

**Background:**

Plastic pollution has become a pervasive global challenge, threatening both aquatic ecosystems and human health. This study explores the biotechnological potential of native microorganisms from diverse aquatic environments for the biodegradation of synthetic plastics and microplastics.

**Methodology and results:**

A total of 200 water samples were collected from freshwater and saltwater sources, yielding 277 bacterial isolates. Preliminary screening showed that approximately one-third of these isolates exhibited plastic-degrading activity, supported by enzymatic functions such as catalase, lipase, protease, esterase, and peroxidase. Seasonal and spatial variations shaped microbial diversity and enzymatic potential, with saltwater habitats harboring the highest diversity. Molecular identification using 16S rRNA gene sequencing revealed that the most efficient degraders were *Micrococcus luteus*, *Enterobacter cloacae*, *Corynebacterium aurimucosum*, and *Mesobacillus maritimus*. Structural and chemical analyses using scanning electron microscopy (SEM) and nuclear magnetic resonance (NMR) provided clear evidence of polymer degradation in both commercial and environmentally collected plastics, with the latter showing greater susceptibility due to pre-weathering. High-performance liquid chromatography (HPLC) further confirmed the presence of plastic-derived contaminants in aquatic samples, particularly in wastewater effluents.

**Conclusion:**

A bacterial consortium composed of confirmed high-efficiency degraders demonstrated remarkable plastic-degrading capacity, highlighting its potential for application in bioremediation strategies within aquatic environments. This consortium was capable of breaking down polyethylene, polyethylene terephthalate, polyhydroxyalkanoates, and low-density polyethylene. These results emphasize the ability of indigenous microbial communities to degrade persistent plastics and underscore their promise for developing eco-friendly bioremediation strategies to mitigate aquatic plastic pollution.

## 1 Introduction

Since the mid-20th century, the rapid growth in plastic production and use has resulted in pervasive environmental contamination. Plastics are manufactured at very large scale because they are cheap to produce, easy to process, and resistant to physical and chemical degradation, but this same persistence causes them to accumulate in the environment once discarded. In 2021, global plastic production exceeded 390 million metric tons, and almost half of this volume was used for short-lived, single-use applications such as packaging materials, plastic bags, beverage bottles, straws, and disposable food-service items (Plastics Europe, 2022). These products are typically used for only a short period before disposal and therefore represent a major component of plastic waste streams. The dominant sources of plastic pollution include mismanaged urban and household waste, industrial discharges and pellet losses, inadequate landfill and waste management practices, the widespread application of plastics in agriculture, and litter associated with recreational and tourism activities, particularly in coastal regions and along rivers and lakes (Jambeck et al., 2015; Hopewell et al., 2009). Together, these pathways introduce large quantities of plastic into terrestrial and aquatic systems, where they accumulate over time and contribute to the growing burden of plastic debris in the environment (Jambeck et al., 2015; Hopewell et al., 2009).

In urban settings, poor waste infrastructure causes plastic to enter rivers and oceans through storm drains. Agricultural practices contribute through degradation of plastic mulching films, greenhouse covers, and drip irrigation components. Industries add to the burden by releasing plastic pellets (nurdles), synthetic fibers, and contaminated effluents into the environment. Furthermore, marine activities such as shipping, aquaculture, and commercial fishing contribute significantly by discarding plastic gear and packaging into the sea (Beaumont et al., 2019). Once released into the environment, plastics are often transported by wind and water into aquatic systems where they accumulate in rivers, estuaries, and oceans. Over time, they fragment into microplastics and nano-plastics, which are ingested by aquatic organisms and become embedded in food chains (Wright and Kelly, 2017). Alarmingly, microplastics have even been detected in human blood and placental tissue, raising serious health concerns (Ragusa et al., 2021). These impacts extend to economic sectors as well, causing damage to fisheries, shipping, and tourism estimated in billions annually (Beaumont et al., 2019).

The environmental consequences of plastic pollution are multifaceted and severe. In marine ecosystems, plastics are ingested by a wide range of organisms, from plankton and fish to seabirds and whales, leading to starvation, internal injuries, and death. Microplastics, formed from the fragmentation of larger debris, have been found in the digestive systems of over 700 marine species, and even in human organs, blood, and placenta, raising serious concerns about their potential toxicity and long-term health effects (Wright and Kelly, 2017; Ragusa et al., 2021). Beyond biological impacts, plastic debris disrupts ecosystem functioning, smothers coral reefs and mangroves, and can transport invasive species and pathogens across oceans. Economically, plastic pollution burdens coastal tourism, fisheries, and shipping, with global damages estimated in billions of dollars per year (Beaumont et al., 2019). Aquatic systems, particularly rivers and coastal areas, act as conduits and sinks for plastic debris. Rivers such as the Nile in Egypt transport tons of plastic waste from inland regions to seas and oceans (Lebreton et al., 2017). In marine environments, plastics not only pose physical hazards to aquatic organisms through ingestion and entanglement but also act as vectors for toxic chemicals and invasive species, further endangering biodiversity (Rochman et al., 2013).

Conventional methods for plastic waste disposal—such as incineration and landfilling—are not sustainable. Incineration emits toxic gases like dioxins and furans, while landfills often leak microplastics and chemical additives into surrounding soil and water bodies (Hopewell et al., 2009). Recycling, though ideal in principle, is hampered by low efficiency, high contamination rates, and poor economic feasibility, especially in low- and middle-income countries (Jambeck et al., 2015). In response to these limitations, biodegradation has emerged as a promising strategy for mitigating plastic pollution. Microorganisms, especially bacteria and fungi, can colonize plastic surfaces (the “plastisphere”) and secreting enzymes such as hydrolases, lipases, cutinases, and esterases that break down complex polymers into metabolizable monomers (Yoshida et al., 2016; Urbanek et al., 2018). Among bacteria, genera like *Pseudomonas*, *Bacillus*, *Micrococcus*, *Enterobacter*, and *Streptomyces* have been frequently identified in studies related to plastic degradation (Singh and Sharma, 2008).

Experimental microbiology of plastic biodegradation tests how microbes attack polymers under controlled conditions. Microorganisms from soil, water, sediments, or bioreactors are isolated or used as consortia and exposed to defined plastic types as carbon sources on solid or liquid media. Researchers monitor growth, biofilm formation on plastic surfaces, clear zones of hydrolysis, changes in OD_600_ and CFU counts, and sometimes mass loss or surface alteration of the polymer. Enzymatic assays for activities such as lipases, esterases, cutinases, and oxidoreductases are used to link degradation to specific biochemical functions. Experimental designs typically manipulate variables such as temperature, pH, salinity, oxygen, and nutrient availability to determine conditions that enhance or limit plastic breakdown and to identify strains or communities with the highest biodegradation potential (Danso et al., 2019). In parallel with experimental microbiology, machine learning and other computational approaches now play an increasing role in environmental monitoring and in the analysis of complex biological datasets. Core work on learning algorithms with limited training data has shown that careful model selection and feature engineering can yield robust classification performance even in small datasets, which is a common constraint in ecological and microbiological studies. More recent studies apply supervised learning, spectral analysis, and remote sensing to detect and quantify plastic and microplastic pollution, and to map spatiotemporal patterns in aquatic systems (Khanam et al., 2025). These developments show that algorithmic tools can complement culture-based and biochemical assays by improving detection sensitivity, supporting automatic classification, and enabling large-scale environmental assessments.

Marine microorganisms have emerged as a promising biological tool in addressing the growing crisis of plastic and microplastic pollution. Unlike physical or chemical remediation methods, microbial degradation offers an eco-friendly and potentially scalable solution to plastic waste that accumulates in marine environments. Certain marine bacteria and fungi have evolved to colonize plastic surfaces, forming what is known as the “plastisphere”, where they secrete enzymes capable of breaking down synthetic polymers into smaller, bioavailable compounds (Zettler et al., 2013). Species belonging to genera such as *Alcanivorax*, *Pseudomonas*, *Vibrio*, and *Bacillus* have been reported to metabolize low-density polyethylene (LDPE), polyethylene terephthalate (PET), and polystyrene (PS) under marine conditions (Urbanek et al., 2018; Danso et al., 2019). The biodegradation process typically involves enzymatic cleavage of long polymer chains followed by microbial assimilation and mineralization into CO_2_ and water. Recent advances in metagenomics and proteomics have facilitated the discovery of novel plastic-degrading enzymes, such as PETase and MHETase, from marine isolates that show activity even at low temperatures and high salinity (Yoshida et al., 2016). Furthermore, marine microbial consortia—rather than individual strains—have demonstrated higher degradation efficiency due to synergistic metabolic interactions (Ru et al., 2020). Beyond plastics, these microorganisms may also play a critical role in microplastic degradation. Given their small size and widespread distribution, microplastics are easily ingested by marine organisms and enter the food web. Marine microbes capable of biofilm formation on microplastic surfaces can alter the chemical structure of these particles, thereby initiating their biodegradation (Amaral-Zettler et al., 2020). Harnessing the metabolic potential of marine microbes, through bioaugmentation or bio-stimulation, represents a promising avenue for sustainable marine bioremediation technologies. Therefore, this study aims to explore the biotechnological potential of indigenous microorganisms isolated from polluted aquatic environments across Egypt for the degradation of plastic and microplastic waste. In light of the escalating global crisis of plastic pollution and the growing evidence supporting the role of marine and freshwater microbes in biodegradation, this research seeks to uncover native bacterial strains with natural capabilities to break down synthetic polymers under environmentally relevant conditions.

## 2 Materials and methods

### 2.1 Study materials

All chemicals and reagents used in this study were of analytical grade. Reagents such as NaCl, CaCl_2_·2H_2_O, MgSO_4_·7H_2_O, KH_2_PO_4_, Na_2_HPO_4_, ethanol, glycerol, crystal violet, and Congo red were sourced from ADWIC (Cairo, Egypt) and Sigma-Aldrich (Germany). Biochemical reagents including Kovac’s reagent, catalase reagent (3% H_2_O_2_), oxidase strips, and urease (CLO) test kits were obtained from Himedia (India), El Nasr Pharmaceutical Co. (Egypt), and Oxoid Ltd. (United Kingdom), following standard microbial testing protocols (Cappuccino and Sherman, 2014). A variety of commercial and custom-prepared media were used for bacterial cultivation and biochemical characterization. Commercial media such as nutrient agar, MacConkey agar, blood agar, EMB, MSA, cetrimide agar, triple sugar iron (TSI), and LB broth were obtained from Lab M Ltd. (United Kingdom), Techno Pharmachem (India), and Himedia (India). Custom media, including mineral salts medium (MSM), phenol red lactose broth, and Congo red agar, were prepared following established methods with slight modifications (Atlas, 2010; Prescott et al., 2002). Biodegradation assays employed polyhydroxyalkanoate copolymers—P(3HB-co-19% 3HV) and P(3HB-co-97% 3HV)—as the sole carbon source. These polymers were emulsified or solvent-dispersed and incorporated into mineral agar for plate-based screening (Pathak and Navneet, 2017).

### 2.2 Sample collection and handling

A total of 200 water samples were collected from various heavily polluted aquatic environments across Egypt (Supplementary Figure S1). Saltwater samples were taken from five sites along the Red Sea (Dahab, Cyrene Island, Ain El-Sokhna, South Sinai, and Hurghada) and five sites along the Mediterranean Sea (Kafr Elsheikh, Port Said, Damietta, Alexandria, and Marina). Sampling followed a transect extending from the shoreline: directly at the beach (Zone A), and at 10 m (Zone B), 20 m (Zone C), and 50 m (Zone D) offshore. At each site and in each season, the order of sampling across zones was randomized to avoid systematic bias due to time of day or tidal state. One sample was collected from each zone per site, totaling 40 samples per season. Seasonal sampling was conducted throughout the year to capture temporal variations in microbial structure and environmental factors, resulting in 160 saltwater samples overall. Freshwater samples were collected from the Nile River and four major canals (Dessok, Ibrahimiya, Mahmoudiyah, and Ismailia). Samples were taken near the shore (Zone A) and from the middle of the river or canal (Zone B), ensuring coverage of spatial variation in microbial distribution. From each site, one sample per zone was collected, giving 10 samples per season. Seasonal repetition brought the total number of freshwater samples to 40. Each combination of site, zone, and season was treated as an independent field replicate. All samples were collected in sterile glass flasks, stored at 4 °C, and processed within 24 h to maintain microbial viability (APHA, 2017). On-site measurements included pH, color, odor, and turbidity. pH values were determined using a calibrated electrometric pH meter at 25 °C.

### 2.3 Isolation and enrichment of aquatic bacteria from polluted freshwater and saltwater

After transport to the laboratory, each water sample was processed within 24 h for enrichment and isolation of bacteria. For saltwater samples, 10 mL of each sample were added to 90 mL of sterile marine nutrient broth prepared from commercial nutrient broth (Lab M, United Kingdom) in artificial seawater adjusted to the *in situ* salinity. For freshwater samples, 10 mL of each sample were added to 90 mL of the same nutrient broth prepared in distilled water with 0.85% NaCl. Enrichment cultures were incubated at 30 °C for 24–48 h with shaking at 120 rpm to favor growth of heterotrophic bacteria. After enrichment, cultures were serially diluted in sterile 0.85% NaCl and spread on a panel of solid media to recover morphologically and physiologically diverse isolates. The following media were used. Nutrient agar for total heterotrophic bacteria. MacConkey agar for Gram-negative enteric and coliform bacteria. Mannitol salt agar for salt tolerant staphylococci and related Gram-positive cocci. Cetrimide agar for *Pseudomonas* like bacteria. Blood agar for fastidious and hemolytic bacteria. EMB and other selective media were used when needed to further differentiate Gram-negative isolates. Plates were incubated at 30 °C–37 °C for 24–72 h. Colonies with distinct morphology were purified by repeated streaking on nutrient agar and stored on nutrient agar slants at 4 °C and in glycerol stocks at −80 °C for subsequent biochemical and plastic degradation assays.

### 2.4 Detection of biodegradative enzymes in candidate plastic-degrading bacterial isolates

To assess the enzymatic capabilities of the candidate plastic-degrading isolates, a series of biochemical assays were performed to detect key enzymes potentially involved in hydrolytic degradation, including catalase, peroxidase, urease, lipase, and citrate utilization. These enzymes are commonly associated with microbial metabolism of complex carbon compounds, including plastics and their byproducts. All tests were performed in triplicate for consistency, and control strains were used to validate the reliability of the biochemical results (Supplementary Table S1). Enzyme activity was converted into a five-point score based on the percentage of detectable reaction intensity. A score of 1 was assigned when the enzyme reaction was detectable at low levels (0–20%). A score of 2 indicated weak detection (21–40%). A score of 3 indicated moderate detection (41–60%). A score of 4 indicated high detection (61–80%). A score of 5 indicated very high detection (81–100%). The same scoring system was applied to all tested enzymes.

#### 2.4.1 Catalase activity test

Catalase activity was tested by adding a few drops of 3% hydrogen peroxide (H_2_O_2_) directly onto a freshly grown colony on a nutrient agar plate or glass slide. The immediate formation of oxygen bubbles indicated a positive catalase reaction, signifying the breakdown of hydrogen peroxide into water and oxygen—a common defense mechanism in aerobic and facultative anaerobic bacteria (Cappuccino and Sherman, 2014).

#### 2.4.2 Peroxidase activity test

Peroxidase activity was most commonly detected using guaiacol agar plates, where guaiacol served as a chromogenic substrate. Microbial isolates were inoculated onto agar medium supplemented with 0.01%–0.04% guaiacol and incubated at 28 °C–37 °C for 24–72 h. Peroxidase-producing colonies oxidized guaiacol in the presence of hydrogen peroxide, resulting in the formation of reddish-brown zones around the colonies. The intensity and diameter of the colored halos were used to assess the relative peroxidase activity. This method was widely applied due to its simplicity, rapid visualization, and sensitivity in screening large numbers of isolates (Kumar et al., 2011; Singh et al., 2017).

#### 2.4.3 Urease test

Urease production was assessed by inoculating a well-isolated colony into 5 mL of urease agar slant (containing urea and phenol red indicator). The tubes were incubated at 37 °C for 4–18 h. A color change from orange to pink indicated a positive urease reaction, as ammonia released by urease activity raises the medium’s pH (Hussain Qadri et al., 1984).

#### 2.4.4 Lipase activity test

Lipase activity was evaluated by streaking isolates onto tributyrin agar plates (or spirit blue agar where applicable), which contain tributyrin oil as a lipid substrate. Plates were incubated at 30 °C–37 °C for 48 h. A clear zone of hydrolysis surrounding the bacterial growth indicated extracellular lipase activity, reflecting the organism’s ability to hydrolyze ester bonds in lipid compounds (Vague et al., 2019). This enzymatic function is of particular interest in plastic degradation due to the structural similarities between ester bonds in lipids and certain plastic polymers like polyesters.

#### 2.4.5 Protease activity test

Protease activity was most commonly detected using skim milk agar (SMA) plates, which contained casein as the primary substrate. Bacterial isolates were spot-inoculated onto SMA and incubated at 30 °C–37 °C for 24–48 h. The hydrolysis of casein by extracellular proteases resulted in a clear halo surrounding the colonies, contrasting with the opaque background of the medium. The diameter of the clear zone, corrected for colony size, was measured to estimate proteolytic activity. This method was widely adopted due to its simplicity, low cost, and reliable visualization of casein hydrolysis (Hankin and Anagnostakis, 1975; Vijayaraghavan and Vincent, 2013).

#### 2.4.6 Esterase activity test

Esterase activity was most effectively detected using tributyrin agar plates, which incorporated the short-chain triglyceride tributyrin emulsified into a nutrient agar base. Test isolates were inoculated and incubated at 25 °C–37 °C for 24–72 h. Active strains produced clear zones around their colonies as tributyrin was hydrolyzed into glycerol and butyric acid, resulting in localized clearing of the otherwise opaque medium. The halo diameter was measured to compare esterase activity levels across isolates. This plate-based method was considered the most straightforward and widely described approach for screening microbial esterases (Samad et al., 1989; Kouker and Jaeger, 1987).

### 2.5 Preliminary screening of plastic degradation

Thirty high-performing aquatic bacterial isolates were selected for further evaluation by choosing two strains from each of 15 sampling sites, comprising 5 Mediterranean Sea sites, 5 Red Sea sites, and 5 Nile and canal sites. High-performing isolates were defined according to the breadth of their enzymatic profile. For each isolate, we recorded the presence or absence of activity for all investigated enzymes. Isolates that showed positivity for the largest number of enzymes in the panel were classified as high performers. This qualitative classification emphasized metabolic versatility and was used to compare isolate performance across seasons, water types, sources, and zones. All assays were performed in a basal mineral medium BMM prepared in deionized water and supplemented with NH_4_Cl 1 g L^−1^, KH_2_PO_4_ 0.5 g L^−1^, MgSO_4_·7H_2_O 0.2 g L^−1^ and trace elements, adjusted to pH 7.6–7.8. For marine isolates, NaCl was added to reach 30–35 PSU. For freshwater isolates, NaCl was omitted to maintain low salinity. The medium contained no added organic carbon so that plastics served as the sole carbon source. All plastic substrates (polyethylene PE, polyethylene terephthalate PET, polystyrene PS, polypropylene PP, and polycaprolactone PCL) were prepared using a single standardized protocol. Sheets and films were cut into discs 6–8 mm in diameter or squares 1 × 1 cm, whereas pellets and powders were weighed directly. All materials were washed in 2% Tween-80, rinsed three times in sterile distilled water, sterilized in 70% ethanol for 20 min, and air-dried under aseptic conditions. Plastics used within the same experiment were always taken from the same pre-washed and sterilized batch to avoid batch-to-batch variation. For agar-based screening, pre-sterilized plastic powders were resuspended in 0.05% Tween-80, dispersed by vertexing and probe sonication, and incorporated into molten BMM agar at the desired concentration. For liquid cultures, plastics were supplied as either emulsified suspensions or solid pieces at 0.5–1% w v or as one disc per vessel.

Inocula were prepared by growing each isolate overnight in BMM containing 0.2% glucose, harvesting the cells, washing twice in carbon-free BMM, and adjusting the cell density to OD_600_ = 0.1. Plastic pieces or emulsified suspensions were randomly assigned to flasks or plates using a random-number list to prevent positional or handling bias. Every experiment included four controls prepared with the same media and plastic batches: sterile BMM plus plastic without cells (abiotic control), BMM with 0.2% glucose (positive growth control), BMM without added carbon (inoculum control), and BMM with emulsifier but no plastic (emulsifier control). All assays were performed using three independent biological replicates (n = 3), each started from a separately prepared overnight culture to ensure true biological replication (Supplementary Table S1). Plastic-degrading potential was assessed using three complementary methods. In the Clear Zone Method CZM, isolates were spot-inoculated (5 µL) onto BMM agar plates containing dispersed plastic (0.1–0.5% w v) and incubated at 25 °C–28 °C for 7–14 days (extended to 28 days for slow growers). Colonies were scored based on halo formation around growth, with clearance index calculated as halo diameter divided by colony diameter (Pranamuda et al., 1995; Gajendiran et al., 2016). In parallel, optical density OD_600_ was monitored in plastic-supplemented liquid BMM to evaluate growth on plastic relative to controls (Supplementary Table S1), with ΔOD_600_ ≥ 0.05–0.1 considered evidence of utilization (Yoon et al., 2012; Shah et al., 2008). Colony-forming units (CFUs) were also quantified by plating serial dilutions at defined intervals, and a ten-fold increase relative to the inoculum control was considered strong evidence of plastic-supported growth (Kathiresan, 2003; Skariyachan et al., 2016). The integration of qualitative halo formation and quantitative OD and CFU measurements provided a multi-parameter evaluation of each isolate’s biodegradation potential.

### 2.6 Molecular identification of candidate plastic-degrading isolates

Genomic DNA was extracted from the most efficient plastic-degrading bacterial isolates using the QIAamp DNA Mini Kit (Qiagen, United States), following the manufacturer’s instructions for Gram-negative and Gram-positive bacteria. Overnight cultures grown in LB broth were centrifuged at 8,000 rpm for 5 min, and the pellets were washed with phosphate-buffered saline (PBS) to remove media components. Bacterial cells were lysed using proteinase K and lysis buffer to ensure efficient release of DNA, particularly in robust cell walls (Wilson, 2001). DNA concentration and purity were measured using a NanoDrop 2000 spectrophotometer (Thermo Scientific, United States), and integrity was assessed *via* 1% agarose gel electrophoresis stained with ethidium bromide. Amplification of the 16S rRNA gene was performed using the universal primers F27 (5′-AGA​GTT​TGA​TCC​TGG​CTC​AG-3′) and R1492 (5′-GGT​TAC​CTT​GTT​ACG​ACT​T-3′), targeting conserved regions common to most bacterial species (Weisburg et al., 1991). Each 25 µL PCR reaction contained 1× PCR buffer, 2.5 mM MgCl_2_, 0.2 mM of each dNTP, 1 µM of each primer, 1.25 U of Taq DNA polymerase (Thermo Fisher Scientific, United States), and 50–100 ng of genomic DNA. The thermocycling profile consisted of an initial denaturation at 95 °C for 5 min, followed by 35 cycles of 95 °C for 30 s, 55 °C for 30 s, and 72 °C for 90 s, with a final extension at 72 °C for 10 min.

The resulting PCR products (∼1.5 kb) were confirmed by electrophoresis on 1% agarose gel and visualized under UV light. Amplified DNA fragments were purified using the QIAquick PCR Purification Kit (Qiagen, United States) to remove residual primers and contaminants. Sequencing was carried out bidirectionally using BigDye Terminator v3.1 Cycle Sequencing Kit (Applied Biosystems, United States) on an ABI 3130 Genetic Analyzer, ensuring high-quality base calls across the full amplicon length (Saitou and Nei, 1987). Raw sequence chromatograms were manually curated and assembled using Chromas and BioEdit software. Clean sequences were submitted to the NCBI BLASTN tool to determine species-level identity by comparing with sequences in the GenBank database. Matches with ≥97% identity and high query coverage were considered reliable for taxonomic classification (Stackebrandt and Goebel, 1994). Multiple sequence alignments were generated using ClustalW, and phylogenetic trees were constructed in MEGA6 software using both the neighbor-joining (NJ) and maximum likelihood (ML) methods (Tamura et al., 2013). To assess the robustness of the phylogenetic groupings, bootstrap analysis was performed with 1,000 replicates. This molecular approach allowed not only for accurate identification of the plastic-degrading bacterial isolates but also provided insights into their evolutionary relationships and potential ecological roles in biodegradation processes.

### 2.7 Detection of environmental plastic pollution in the aquatic samples using HPLC

Detection of environmental plastic pollution in aquatic samples was performed using High-Performance Liquid Chromatography (HPLC), a sensitive chromatographic technique capable of identifying and quantifying plastic degradation products and additives such as monomers, oligomers, phthalates, and bisphenols at the molecular level. Five fresh water sampling was carried out at the wastewater treatment plant (WWTP) final effluent and in the adjacent downstream mixing zone, which were selected as representative hotspots for plastic pollution. For comparison with freshwater samples, five saltwater samples were collected from the coastal areas adjacent to a marina, and from the seashore. Water samples suspected of contamination were first subjected to extraction and filtration to isolate potential degradation compounds. The extracts were then analyzed by HPLC using reverse-phase C18 columns under gradient elution conditions. Detection was performed using UV-Vis spectroscopy at appropriate analytical wavelengths (220–280 nm, depending on compound). Quantification was achieved by comparison with calibration curves prepared from plastic-related reference standards, including terephthalic acid (TPA) for polyethylene terephthalate (PET), bisphenol A (BPA) for polycarbonate, and Di(2-ethylhexyl) phthalate (DEHP), Dibutyl phthalate (DBP) and Dimethyl phthalate (DMP) for phthalates as plasticizer markers (Hermabessiere et al., 2017; Enfrin et al., 2019). This analytical approach provided accurate and reproducible evidence of plastic-derived compounds in aquatic environments, thereby enabling a clearer understanding of contamination levels and supporting ecological risk assessment and remediation strategies. Analyses were conducted at the Micro Analytical Center, Cairo University, and compared against known standards (Mohana et al., 2008).

### 2.8 Plastic and microplastic sources for biodegradability analysis

Plastic substrates used for biodegradability testing were obtained from both commercial and environmental sources to simulate real-world contamination scenarios and allow comparative analysis of microbial degradation across different polymer types. Commercial plastic samples included standardized forms of synthetic polymers such as low-density polyethylene (LDPE), polyhydroxyalkanoates (PHA), and polyethylene terephthalate (PET). LDPE and PET films were purchased from Sigma-Aldrich (United States) and used as model substrates. Commercial polyethylene-based microplastic beads (50–500 µm in diameter) were also used for standardized comparison. In addition, environmental plastic and microplastic samples were collected from heavily polluted freshwater and marine locations across Egypt, including the Nile River, agricultural canals, Dahab Beach, Alexandria, Port Said, and Marina. These included visibly degraded materials such as plastic bags, bottle caps, packaging films, and fishing gear fragments, which represent the primary components of plastic waste in natural water bodies (GESAMP, 2015). Samples were thoroughly washed with distilled water, air-dried, sterilized in 70% ethanol, and exposed to ultraviolet light for 30 min per side before experimental use (Shah et al., 2008). To produce lab-grade microplastic from field or commercial waste, selected plastics were cryogenically ground and sieved to generate particles smaller than 5 mm, aligning with the standard microplastic definition proposed by international environmental agencies (GESAMP, 2015). All macro- and microplastic materials were used as the sole carbon source in mineral salts medium (MSM) and liquid carbon-free medium (LCFM) during microbial biodegradability assays, following previously validated protocols (Shah et al., 2008; Pathak and Navneet, 2017).

### 2.9 Integrated structural and chemical analysis of plastic biodegradability using SEM and NMR

#### 2.9.1 Consortium assembly of plastic-biodegrading isolates

For the evaluation of biodegradation activity, a defined bacterial consortium was prepared by combining the four isolates that exhibited the highest and most consistent plastic-degrading potential. Each isolate was first pre-cultured individually in standard nutrient broth (Oxoid, United Kingdom; 5 g L^−1^ peptone, 3 g L^−1^ beef extract, 5 g L^−1^ NaCl) at 30 °C with shaking at 150 rpm until mid-exponential phase (OD_600_ ≈ 0.6–0.8). To minimize carry-over of organic nutrients into the plastic degradation assays, cells were harvested by centrifugation (5,000–8,000 × g, 10 min), washed at least twice in carbon-free BMM, and finally resuspended in fresh carbon-free BMM. For each isolate, an OD–CFU relationship was calibrated by measuring OD_600_ of washed cell suspensions and determining CFU mL^−1^ by plate counts on BMM agar supplemented with 0.2% glucose; these isolate-specific calibrations were used to adjust each culture to OD_600_ = 1.0, corresponding to approximately 1 × 10^8^ CFU mL^−1^. Equal volumes (5 mL) of each standardized suspension were then combined to obtain a 20 mL mixed consortium in which all four strains contributed comparable CFU numbers to the inoculum. This washed and CFU-standardized consortium was used to inoculate plastic-containing media, reducing the risk that early growth was driven by residual nutrients rather than by compounds released from the commercial and environmental plastic substrates (Restrepo-Flórez et al., 2014). For each experimental run, the consortium was prepared fresh using the same standardization procedure, and the same consortium batch was used to inoculate all replicate flasks and their corresponding controls, which limited variation in inoculum composition between treatments.

#### 2.9.2 Experimental design for plastic degradation

All plastic samples were cut into uniform coupons, thoroughly washed, ethanol-sterilized, and UV-irradiated before being transferred into sterile 250 mL Erlenmeyer flasks containing 100 mL of carbon-limited medium. Bushnell–Haas medium was used for freshwater samples, and artificial seawater medium was used for saltwater experiments (Shah et al., 2008; Muhonja et al., 2018). Each flask was supplied with a single plastic coupon and inoculated with 5% v v of the bacterial consortium, while appropriate controls (Supplementary Table S1) were maintained, including uninoculated plastics, consortium without plastics, and sterilized plastics only. For each plastic type and water matrix, at least three independent flasks were set up per condition to provide biological replication. Plastic coupons were drawn from the same sterilized batch for a given experiment and were randomly assigned to inoculated and control flasks to avoid systematic bias related to coupon handling or position in the incubator. Abiotic contributions to surface damage and chemical release were evaluated by processing uninoculated plastic controls under exactly the same incubation conditions as inoculated flasks, including temperature, shaking, and duration. These control plastics were subjected to the same SEM and NMR workflows as the inoculated plastics, and only changes that were absent or minimal in abiotic controls but pronounced in inoculated samples were interpreted as evidence of microbial degradation rather than abiotic weathering. For environmental plastics, an additional time zero set of coupons was documented by SEM and NMR immediately after cleaning and sterilization, before inoculation, to record pre-existing weathering features and chemical signatures.

Incubations were carried out at 30 °C with shaking at 120 rpm for 14–28 days. During this period, plastic samples were periodically removed for analysis by Scanning Electron Microscopy (SEM), and culture supernatants were examined using Nuclear Magnetic Resonance (NMR) to detect characteristic biodegradation products such as terephthalic acid TPA, bisphenol A BPA, and phthalates including di(2-ethylhexyl) phthalate DEHP, dibutyl phthalate DBP, and dimethyl phthalate DMP.

#### 2.9.3 Assessment of plastic biodegradation

The biodegradability of plastic was assessed using both commercial plastic samples and environmentally collected plastic samples one freshwater and one saltwater through advanced analytical techniques, including Scanning Electron Microscopy SEM and Nuclear Magnetic Resonance NMR spectroscopy. SEM was employed to observe the surface morphology of the plastic samples before and after microbial degradation. Clear evidence of biodegradation, such as cracks, pits, surface erosion, and microbial colonization, was visible on treated samples, particularly in those exposed to high-performing bacterial isolates (Shah et al., 2008; Ojha et al., 2017). For both commercial and environmental plastics, SEM imaging was performed on sterilized coupons prior to incubation, which allowed us to distinguish pre-existing defects due to environmental exposure from new defects that appeared only after incubation with the consortium. Only increases in the extent or intensity of surface cracking, pitting, biofilm coverage, or erosion relative to both time zero images and abiotic controls were attributed to microbial activity.

These structural changes indicated active microbial interaction and breakdown of the polymer matrix, consistent with previous reports on bacterial plastic degradation (Muhonja et al., 2018). Complementing these findings, NMR spectroscopy was used to analyze the chemical structure of the plastic materials, providing molecular-level insights into polymer degradation (Ojha et al., 2017). Shifts or changes in characteristic peaks within the NMR spectra revealed alterations in chemical bonds and the breakdown of polymer chains, confirming microbial-induced depolymerization (Restrepo-Flórez et al., 2014). For each plastic category, spectra from inoculated samples were compared directly to spectra from matched abiotic controls and to time zero extracts, and only new or markedly enhanced signals in the inoculated condition were interpreted as biodegradation products. Environmental plastics often showed more pronounced post-incubation changes than commercial plastics, but this interpretation was based on the magnitude of change within each plastic type relative to its own baseline rather than on absolute differences between plastic types, which helped to account for the higher degree of pre-conditioning and prior oxidation in field-collected materials. We therefore interpret the stronger degradation features observed in environmental plastics as the combined effect of prior environmental weathering and consortium activity rather than as evidence of intrinsically higher microbial efficiency on those substrates.

Together, SEM and NMR provided a comprehensive evaluation of physical and chemical degradation, offering robust confirmation of plastic biodegradability in both controlled commercial samples and naturally weathered environmental plastics.

### 2.10 Statistical analysis

All statistical analyses were performed in software (*SPSS v.29.0*). Continuous variables such as optical density OD_600_, CFU counts, and halo diameters are reported as mean ± standard deviation SD of at least three independent biological replicates n = 3, unless stated otherwise. The normality of residuals and homogeneity of variances were checked using the Shapiro–Wilk and Levene tests. When assumptions were met, we used one-way or two-way analysis of variance ANOVA followed by Tukey’s *post hoc* test to compare means between groups season, source, water type, zone, plastic type. When assumptions were not met, we used the non-parametric Kruskal–Walli’s test with Dunn’s *post hoc* comparisons. Categorical data such as Gram reaction, cell morphology, presence or absence of enzyme activity, and proportion of positive degraders were analyzed using chi-square χ^2^ tests or Fisher’s exact test when expected frequencies were <5. Correlations between enzyme activity scores and plastic degradation performance CZM halo, OD_600_ increase, CFU increase were evaluated using Spearman’s rank correlation coefficient ρ. To explore multivariate patterns, we performed principal component analysis PCA on centered and scaled variables including enzyme activities, water type, source, zone, and degradation metrics, and visualized sample clustering in ordination plots. All tests used a significance threshold of p < 0.05. Moreover, Correlation analysis to detect the correlation coefficient (*r-value*) was performed in *R-program v4.3.2* on the 30 isolates tested in all three degradation assays. A data frame was created with one row per isolate and numeric columns for Gram reaction, morphology, enzyme activities, habitat (fresh/salt), season, and degradation metrics CZM(HF), CZM(OD), and CZM(CFU); binary traits were coded as 0 negative and 1 positive, and OD_600_ and CFU values were entered as the mean of three independent experiments n = 3.

## 3 Results

### 3.1 Preliminary microbiological screening

**FIGURE 1:**
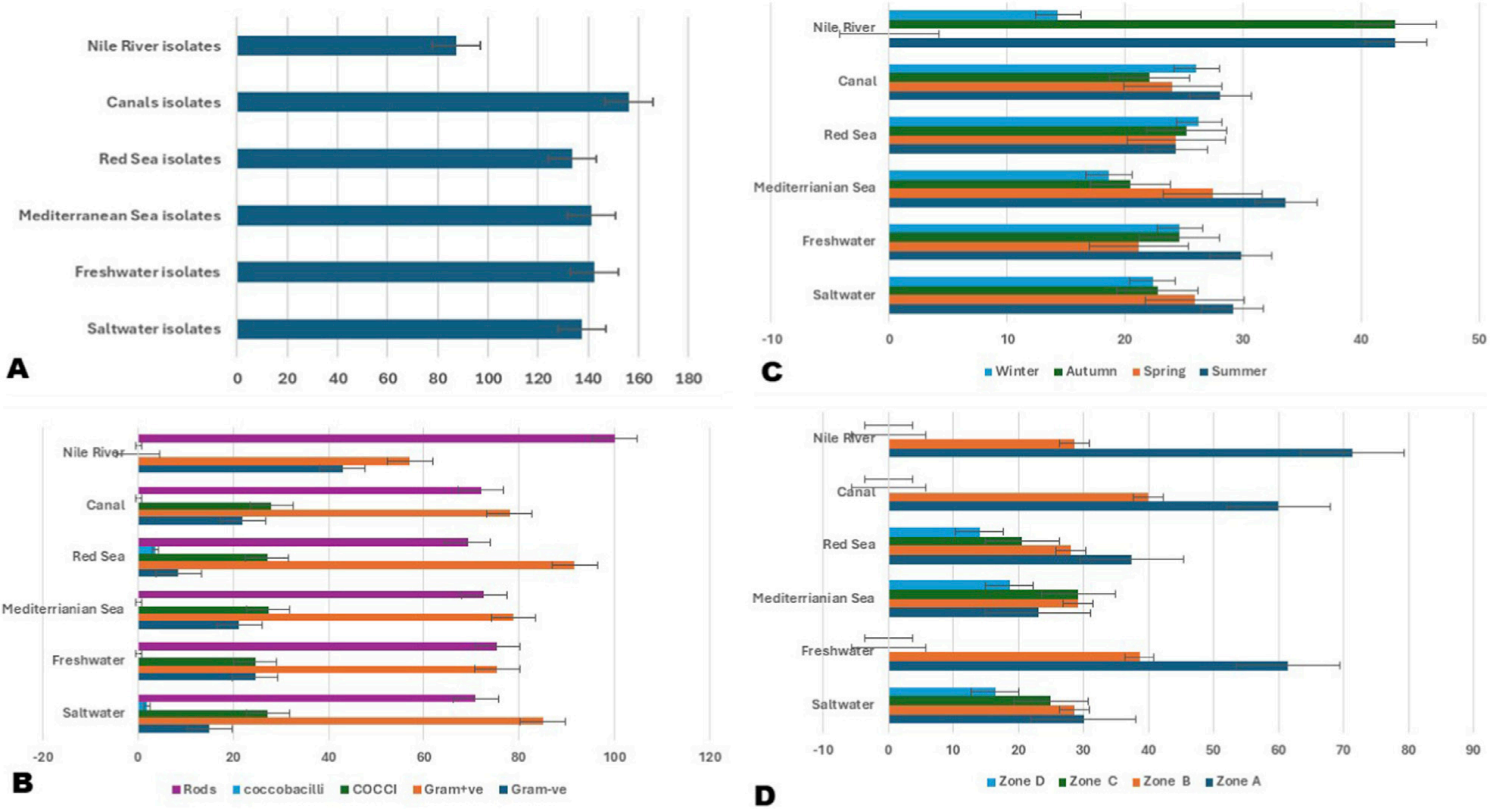
Distribution and characteristics of bacterial isolates recovered from different aquatic sources. **(A)** Total number of bacterial isolates obtained from saltwater (Mediterranean Sea, Red Sea) and freshwater (Canals, Nile River) sources. **(B)** Morphological and Gram staining characteristics of isolates (rods, cocco-bacilli, cocci, Gram-positive, Gram-negative) across sampling sites. **(C)** Seasonal distribution of isolates in different aquatic environments during winter, spring, summer, and autumn. **(D)** Spatial distribution of isolates across sampling zones **(A–D)** within each aquatic source. Error bars indicate standard deviations.

Out of the 200 collected water samples, 178 (89%) showed positive microbial growth with a total of 277 recovered isolates (Table 1). The majority of the investigated samples supported growth of only one isolate, with progressively fewer samples supporting multiple isolates, which reflected a gradient of microbial diversity across the studied environments. Of note, 22 samples (11%) showed no detectable growth and yielded no isolates. Among the positive samples, 120 (67.4%) harbored a single isolate, 30 (16.8%) contained two isolates (60 in total), 18 (10.1%) contained three isolates (54 in total), 7 (3.9%) contained four isolates (28 in total), and 3 (1.7%) harbored five isolates (15 in total). In contrast, the 22 negative samples did not yield any isolates (Table 1). The distribution of the number of isolates per sample differed significantly from a uniform distribution χ^2^ test, p < 0.05, confirming that most samples harbored only one dominant colony type while multi-isolate samples were less frequent. The prevalence rates (number of isolates/total number of samples) for all sample types exceeded 100%, except for the Nile River samples (Figure 1A).

**TABLE 1:** Summary of sample positivity and isolate distribution in saltwater and freshwater environments.

Category	Count/Total number of samples	Percentage (%)
Positive samples	178/200	89
Positive saltwater samples	146/160	91.2
Positive freshwater samples	32/40	80
Negative samples	22/200	11
Positive samples with 1 isolate	120/178	67.4
Positive saltwater samples with 1 isolate	99/146	67.8
Positive freshwater samples with 1 isolate	21/32	65.6
Positive samples with 2 isolates	30/178	16.8
Positive saltwater samples with 2 isolates	27/146	18.5
Positive freshwater samples with 2 isolates	3/32	9.4
Positive samples with 3 isolates	18/178	10.1
Positive saltwater samples with 3 isolates	15/146	10.3
Positive freshwater samples with 3 isolates	3/32	9.4
Positive samples with 4 isolates	7/178	3.9
Positive saltwater samples with 4 isolates	3/146	2
Positive freshwater samples with 4 isolates	4/32	12.5
Positive samples with ≥5 isolates	3/178	1.7
Positive saltwater samples with ≥5 isolates	2/146	1.4
Positive freshwater samples with ≥5 isolates	1/32	3.1
Total number of isolates	277/200	138.5
Saltwater isolates	220/160	137.5
Freshwater isolates	57/40	142.5
Mediterranean sea isolates	113/80	141.2
Red sea isolates	107/80	133.7
Canals isolates	50/32	156.2
Nile river isolates	7/8	87.5

Gram staining showed that Gram-positive bacteria dominated across all sample types (Figure 1B) with an overall percentage of 83.4% (231 isolates), while Gram-negative bacteria accounted for 16.6% (46 isolates). Morphological analysis revealed that rods were the most prevalent form, representing 72.6% (201 isolates), followed by cocci at 26.7% (74 isolates), and a small fraction of coccobacilli at 0.7% (2 isolates). Gram-positive isolates were significantly more frequent than Gram-negative isolates across all water types χ^2^ test, p < 0.001. Rods were also significantly more common than cocci and coccobacilli χ^2^ test, p < 0.001. The proportion of Gram-negative isolates was higher in saltwater than freshwater samples, although Gram-positive rods still dominated in both environments χ^2^ test, p < 0.05.

The results indicated a clear predominance of Gram-positive rods and cocci. Saltwater environments contained both Gram-positive and Gram-negative bacteria with different shapes, while freshwater samples were mostly Gram-positive cocci and rods, which showed a simpler and more uniform structure (Figure 1B). Of note, the highest proportion of isolates was recovered during the summer season across all sample types, except for the Red Sea samples, where most isolates were obtained in winter (Figure 1C). Of note, the number of isolates differed significantly between seasons χ^2^ test or one-way ANOVA on counts per sample, p < 0.05, with summer yielding more isolates than winter. Across all sample types, most isolates were obtained from Zone A (directly at the beach), whereas in the Mediterranean Sea, Zones B and C yielded the highest numbers. In contrast, Zone D consistently had the fewest bacterial isolates (Figure 1D). Isolate abundance also decreased significantly with distance from the shoreline one-way ANOVA or χ^2^ test across zones A–D, p < 0.01, supporting a nearshore enrichment of culturable bacteria.

### 3.2 Clustering analysis of saltwater and freshwater isolates

**FIGURE 2:**
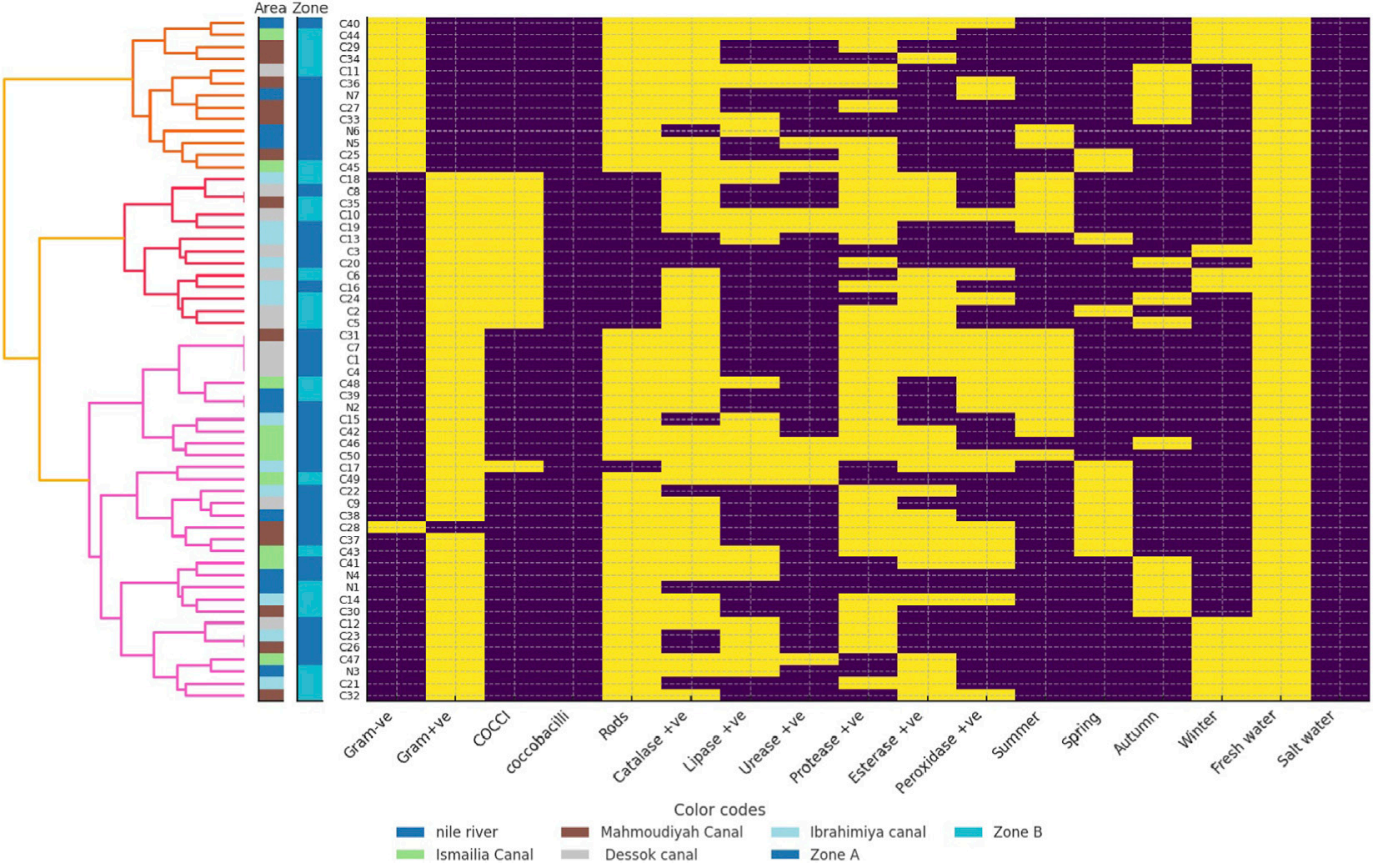
Hierarchical clustering heatmap of freshwater bacterial isolates showing biochemical and environmental characteristics. Each row represents a bacterial isolate labeled on the left, where N denotes isolates from the Nile River and C denotes isolates from canals. Columns correspond to tested traits, including morphological features such as Gram reaction and cell shape, enzyme activities including catalase, lipase, protease, esterase, and peroxidase, seasonal occurrence, and environmental preference freshwater *versus* saltwater. The dendrogram on the left illustrates hierarchical relationships among isolates based on similarity in biochemical profiles. Isolates positioned closer together share more similar trait patterns. Heatmap colors use yellow for a positive response and violet for a negative response. The area strip in the leftmost color bar uses different colors for sampling locations, including the Nile River code (N) and canals code (C) such as Mahmoudiyah Canal, Ismailia Canal, Dessok Canal, and Ibrahimiya Canal. The zone strip in the adjacent color bar uses different colors for ecological zones such as Zone A and Zone B.

**FIGURE 3:**
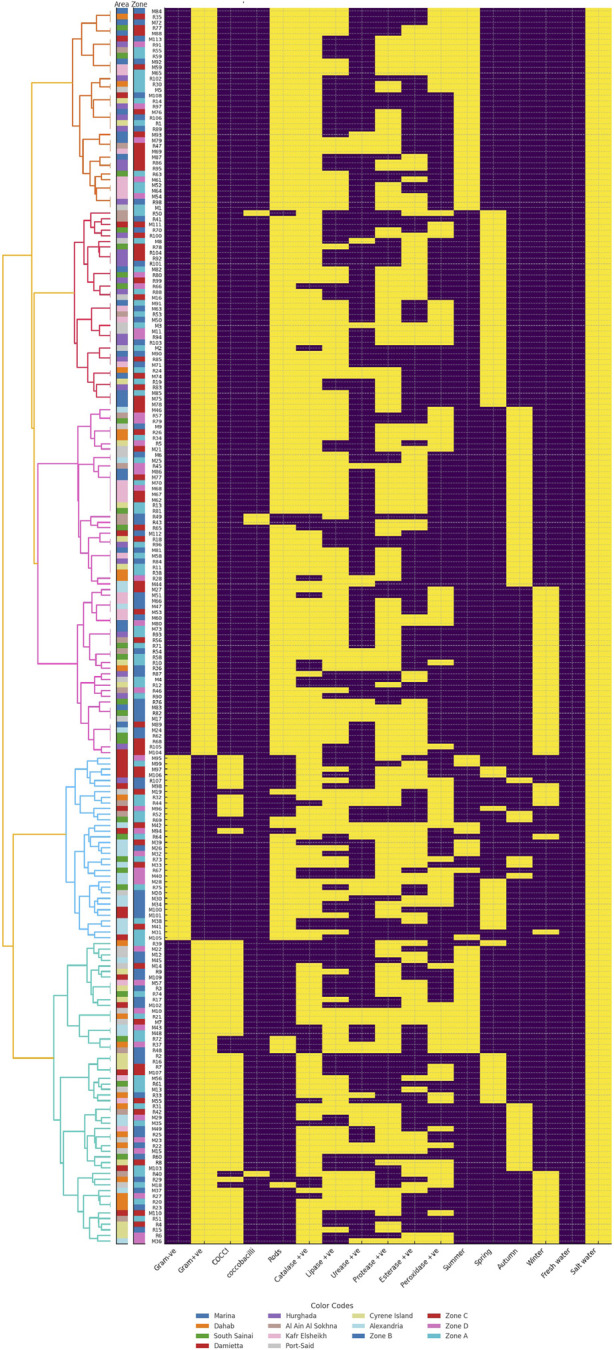
Hierarchical clustering heatmap of saltwater bacterial isolates showing biochemical and environmental characteristics. Each row represents a bacterial isolate labeled on the left, where M denotes isolates from the Mediterranean Sea and R denotes isolates from the Red Sea. Columns correspond to tested traits, including morphological features such as Gram reaction and cell shape, enzyme activities including catalase, lipase, protease, esterase, and peroxidase, seasonal occurrence, and environmental preference freshwater *versus* saltwater. The dendrogram on the left shows hierarchical relationships among isolates based on similarity in biochemical profiles. Isolates that appear closer together share more similar trait patterns. Heatmap colors use yellow for a positive response and violet for a negative response. The area strip in the leftmost color bar uses different colors for sampling locations, including Red Sea sites Dahab, Cyrene Island, Ain El-Sokhna, South Sinai, and Hurghada coded as (R) and five sites along the Mediterranean Sea Kafr Elsheikh, Port Said, Damietta, Alexandria, and Marina coded as (M). The zone strip in the adjacent color bar uses different colors for ecological zones (Zone A, B, C, D).

The clustering of freshwater and saltwater bacterial isolates showed both similarities and differences in their biochemical and ecological patterns (Figures 2, 3). Freshwater isolates from the Nile River and canals showed some overlap between sites, suggesting that related bacterial types occur across connected water systems. In contrast, saltwater isolates from coastal and marine sites were more clearly separated, indicating stronger environmental influence. Most freshwater isolates were Gram-positive rods with high catalase, lipase, and protease activity, showing metabolic flexibility adapted to changes in nutrients and oxygen levels. Their seasonal grouping, during summer and winter, suggested the ability to withstand shifts in temperature and water flow. Saltwater isolates were mainly Gram-positive rods and cocci with strong catalase and lipase activity but lower protease levels than the freshwater isolates. These traits reflected better resistance to oxidative stress and efficient use of lipids, which are useful in saline and changing marine conditions. The more distinct clustering of saltwater isolates indicated clearer separation by site and zone, shaped by salinity and differences in local habitats.

### 3.3 Biochemical activity

#### 3.3.1 Enzymatic biochemical activity of the bacterial isolates

Based on the biochemical activity results of the 277 bacterial isolates, the majority were catalase-positive (85.2%, 236 isolates), while only 14.9% (41 isolates) were catalase-negative. Lipase activity was detected in 58.5% (162 isolates), with 41.6% (115 isolates) lacking this activity. Urease activity was less common, observed in only 22.1% (61 isolates), whereas 78% (216 isolates) were urease negative. Protease activity was prevalent, with 70.4% (195 isolates) testing positive and 29.7% (82 isolates) negative. Esterase activity was nearly evenly distributed, with 49.9% (138 isolates) positive and 50.2% (139 isolates) negative. Lastly, peroxidase activity was present in 38.7% (107 isolates), while the majority (61.4%, 170 isolates) showed no activity (Figures 2, 3). The frequencies of the six enzyme activities differed significantly from each other χ^2^ test, p < 0.001, with catalase and protease occurring at significantly higher proportions than urease and peroxidase *post hoc* pairwise comparisons, p < 0.05.

#### 3.3.2 Biochemical activity and seasonal variations

**FIGURE 4:**
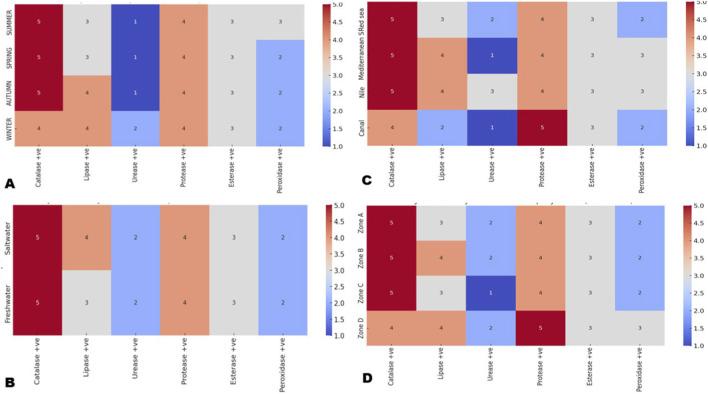
Biochemical activity of bacterial isolates across environmental gradients. Semi-quantitative enzyme activity scores (1–5) were assessed in relation to **(A)** seasons **(B)** water types, **(C)** aquatic sources, and **(D)** spatial zones. Catalase and protease remained constitutively high across all conditions, while urease (low) and esterase (intermediate) showed stable activity. Lipase and peroxidase exhibited pronounced variability: lipase peaked in autumn/winter, saltwater, Nile/Mediterranean, and offshore Zone D; peroxidase remained generally low but increased slightly in Mediterranean samples and Zone D. Seasonal and spatial patterns reflected higher microbial recovery in warmer months, saltwater habitats, and nearshore zones.

Out of the 277 bacterial isolates recovered, summer samples accounted for the highest proportion with 81 isolates (29.3%), followed by spring with 69 isolates (25%), autumn with 64 isolates (23.2%), and winter with 63 isolates (22.8%). This seasonal distribution shows that microbial recovery was slightly higher during the warmer months (summer and spring) compared to the cooler seasons (autumn and winter). Seasonal changes were reflected in the semi-quantitative enzyme activity scores of all detected aquatic bacteria (Figure 4A). Seasonal variation did not affect the scores of protease and esterase, which remained constant in all seasons at 4 and 3, respectively. In contrast, the other enzymes showed seasonal shifts, with summer samples exhibiting the highest biochemical activities, with a score range of 3–5 for the investigated enzymes. Winter samples showed lower scores for most enzymes, but urease activity deviated from this pattern, as it was higher in winter than in summer, indicating that urease responded differently to seasonal conditions than the other measured enzymes. Seasonal differences in total isolate numbers were statistically significant χ^2^ test, p < 0.05. For enzyme activity scores, a two-way ANOVA or Kruskal–Wallis test with season as factor showed that lipase and peroxidase activities varied significantly between seasons p < 0.05, whereas catalase, protease, esterase, and urease did not show significant seasonal variation p > 0.05. These patterns suggest that these enzymes play less prominent roles in seasonal microbial metabolic processes.

#### 3.3.3 Biochemical activity and water types

Among the 277 bacterial isolates, 220 (79.5%) were recovered from saltwater environments, whereas 57 (20.6%) were obtained from freshwater sources. This indicates that saltwater habitats harbored a higher proportion of bacterial isolates compared to freshwater environments. The comparison of enzyme activity between freshwater and saltwater environments (Figure 4B) revealed consistent patterns in microbial enzymatic responses. Saltwater samples showed high biochemical activity, with catalase reaching the maximum score of 5, protease 4, esterase 3, peroxidase 2, urease 2, and lipase showing a relatively higher score of 4. Freshwater samples exhibited a very similar pattern, with catalase 5, protease 4, esterase 3, peroxidase 2, and urease 2, but lipase was lower with a score of 3. Saltwater samples yielded significantly more isolates than freshwater samples when normalized per sampling site t-test or χ^2^ test on counts per sample, p < 0.01. Lipase activity scores were significantly higher in saltwater isolates than in freshwater isolates Mann–Whitney test, p < 0.01, while catalase, protease, esterase, urease, and peroxidase scores did not differ significantly between the two water types p > 0.05.

#### 3.3.4 Biochemical activity and source

Of the 277 bacterial isolates, the Mediterranean Sea contributed the highest number (113 isolates), followed by the Red Sea (107 isolates). In contrast, canals yielded 50 isolates, while the Nile River showed the lowest recovery, with only 7 isolates (Table 1). This distribution shows that marine environments (Mediterranean and Red Sea) were the dominant sources of aquatic bacterial isolates compared to freshwater sources (canals and Nile). Analysis of enzyme activity scores highlighted source-specific patterns in microbial functional potential across aquatic environments (Figure 4C). Mediterranean isolates showed high biochemical activity, with catalase 5, lipase and protease 4, esterase and peroxidase 3, and the lowest urease score of 1. Red Sea isolates had a similar pattern, with catalase and protease scoring 5 and 4, moderate lipase, esterase, and peroxidase scores of 3, 3, and 2, and low urease activity with a score of 2. Nile isolates combined high catalase and protease scores of 5 and 4 with lipase 4, urease 3, esterase 3, and peroxidase 3. Canal isolates differed slightly, with lower catalase 4 and lipase 2, the highest protease score of 5, low urease 1, esterase 3, and peroxidase 2. The number of isolates per source differed significantly by one way ANOVA or χ^2^ test on isolate counts per source, p < 0.001, whereas median enzyme activity scores showed only small differences among Red Sea, Mediterranean, Nile, and canal isolates and none of these differences were significant in Kruskal–Wallis tests, p > 0.05 for all enzymes.

#### 3.3.5 Biochemical activity and zone

Out of the 277 bacterial isolates, Zone A (shoreline/beach) yielded the highest number with 101 isolates (36.5%), followed by Zone B (10 m offshore) with 85 isolates (30.7%). Further offshore, the numbers decreased, with Zone C (20 m offshore) contributing 55 isolates (19.9%) and Zone D (50 m offshore) yielding the lowest at 36 isolates (13%). This distribution indicates a gradual decline in bacterial recovery with increasing distance from the shoreline, suggesting higher microbial abundance and activity in nearshore environments compared to offshore zones. The spatial distribution of enzyme activity across different zones (Figure 4D) revealed both uniform patterns and localized variations in microbial metabolic potential. Zone A, B, and C showed very similar enzyme activity patterns, all dominated by high catalase (score 5) and high protease (scores 4), with moderate lipase and esterase activity and low peroxidase. These three zones also showed low urease activity, with Zone C showing the lowest level. Zone D differed from the others by having slightly lower catalase activity and the slightly higher protease and peroxidase activity among all zones. Overall, Zones A, B, and C were largely comparable, while Zone D displayed a more distinct enzyme activity profile. Isolate abundance decreased significantly from Zone A to Zone D (one-way ANOVA or χ^2^ test, p < 0.01). Lipase, protease, and peroxidase activity scores differed significantly between zones (Kruskal–Wallis test, p < 0.05), with lipase scores higher in Zones B and D, protease highest in Zone D, and peroxidase also highest in Zone D, while catalase and esterase scores remained similar across zones and showed no significant zonal differences (p > 0.05).

### 3.4 Preliminary screening of plastic degradation

**FIGURE 5:**
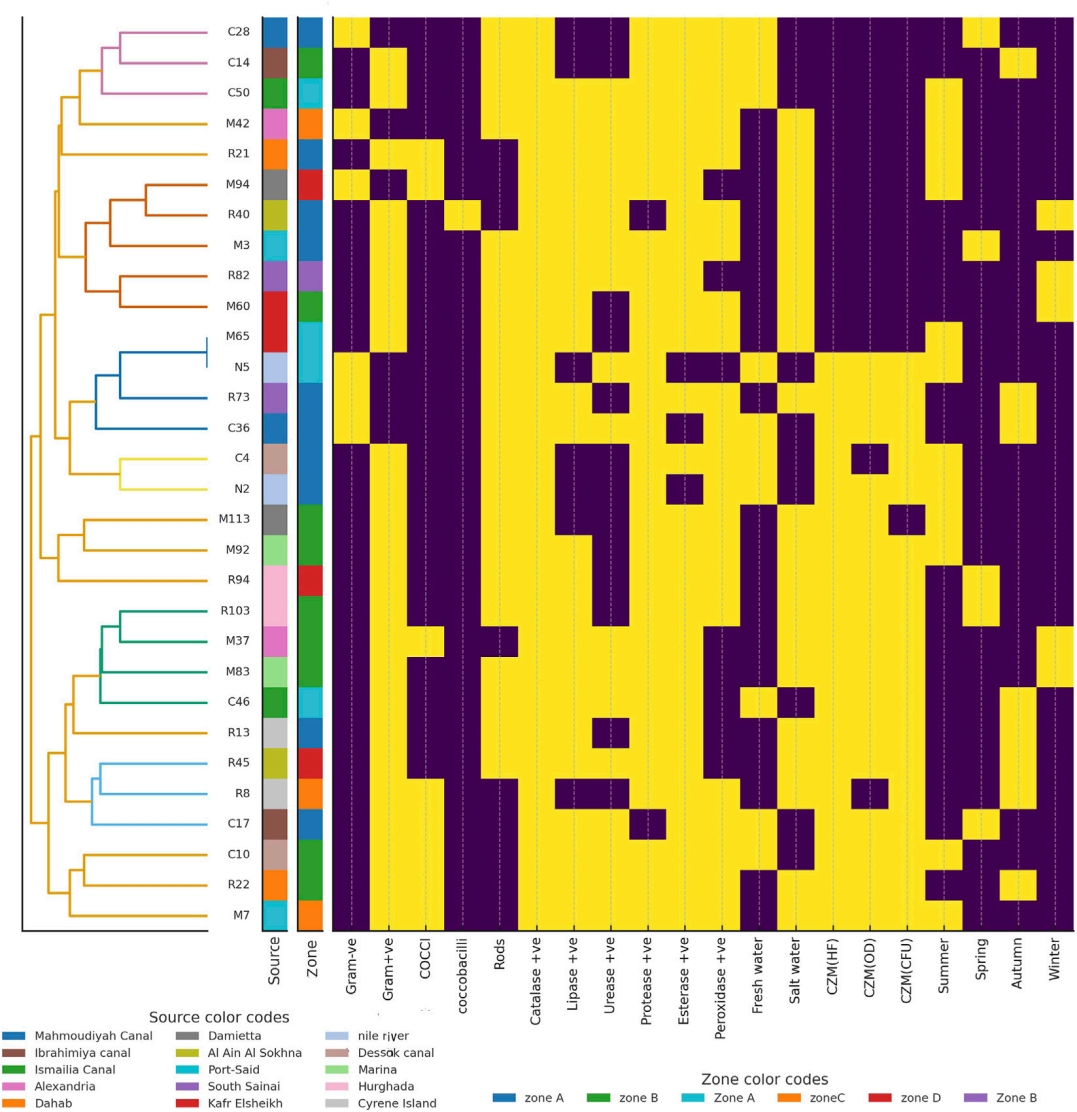
Hierarchical clustering heatmap of the selected 30 bacterial isolates for preliminary screening of plastic degradation based on Phenotypic and biochemical characteristics. Rows represent individual isolates ordered according to the dendrogram on the left and columns represent the scored phenotypic and functional traits. Yellow indicates presence or positive activity value 1 and purple indicates absence or negative activity value 0. The vertical-colored bars next to the dendrogram show the sampling source and spatial zone for each isolate. The “Source color codes” key identifies the coastal and freshwater locations from which isolates were obtained. The “Zone color codes” key identifies the sampling zones within each water body.

Out of the selected 30 isolates tested, 11 (36.7%) produced distinct halos on plastic-containing agar plates in the Clear Zone Method (CZM), indicating visible polymer degradation. In liquid culture assays, 9 isolates (30%) demonstrated optical density (OD_600_) values greater than 0.5, reflecting significant growth and potential utilization of plastic substrates. Furthermore, 10 isolates (33.3%) achieved a ten-fold increase in colony-forming units (CFUs) compared to the baseline or carbon-free controls, providing strong evidence of sustained cell proliferation in response to plastic. For the 30 selected isolates, mean halo diameters, OD_600_ increases, and CFU fold-changes are reported as mean ± SD from three independent experiments n = 3. Isolates that were positive in all three assays showed significantly larger halo diameters, higher OD_600_ and higher CFU increases than isolates positive in zero or one assay one-way ANOVA with Tukey’s *post hoc* test, p < 0.01. Collectively, these findings suggest that only 8 isolates consistently exhibited positive activity across all three assays, highlighting their potential as true plastic-degrading candidates (Figure 5; Supplementary Table S2).

### 3.5 Correlation analysis

**FIGURE 6:**
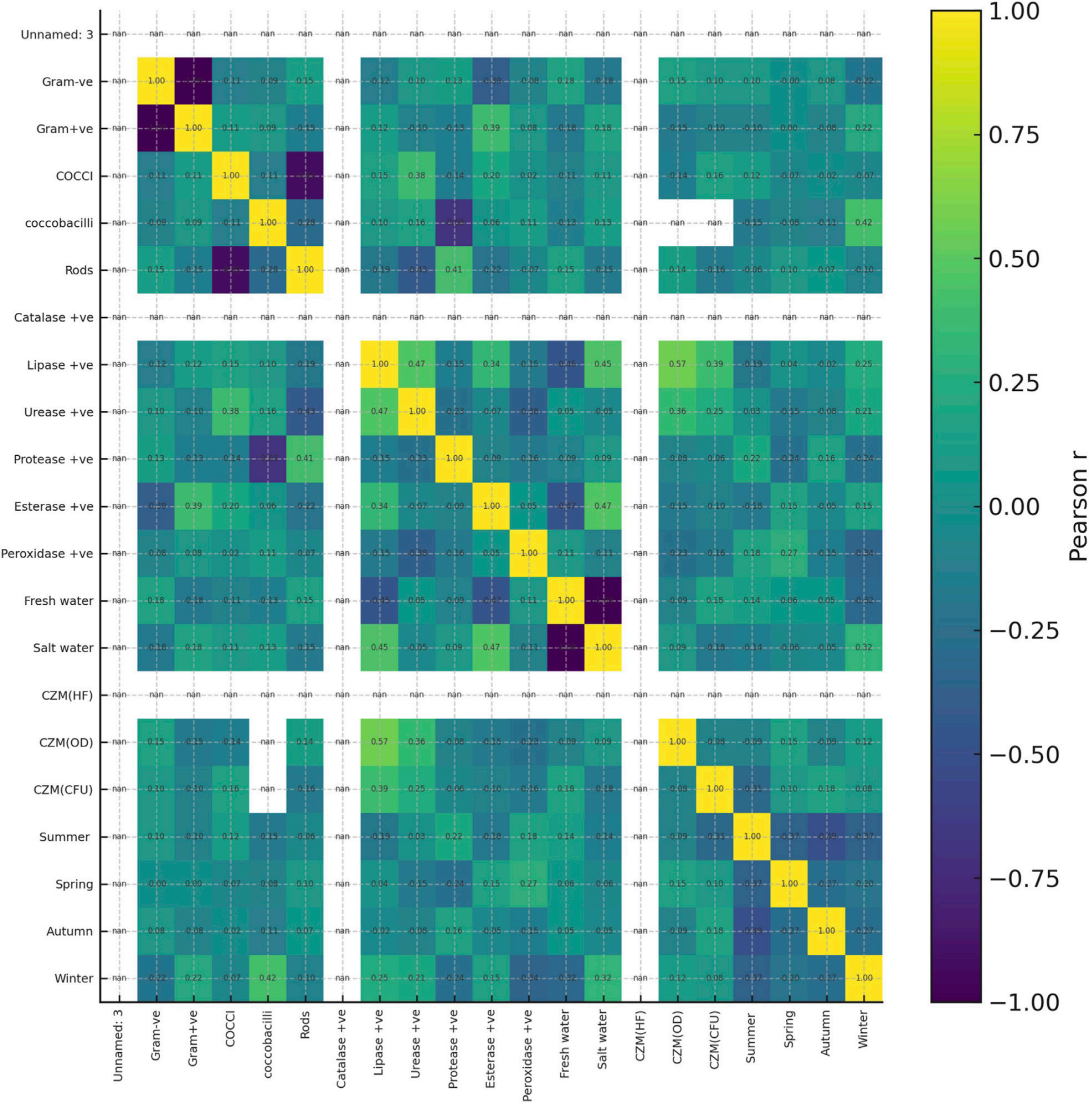
Heatmap showing Pearson correlation coefficients among phenotypic traits and plastic-degradation metrics for the 30 selected isolates. Variables include Gram reaction, cell morphology, enzyme activities catalase, lipase, urease, protease, esterase, peroxidase, habitat fresh water, salt water, seasonal occurrence summer, spring, autumn, winter, and degradation readouts CZM(HF), CZM(OD), and CZM(CFU). Colors indicate the strength and direction of the correlation from −1 negative, blue to +1 positive, yellow as shown in the scale bar on the right. Numeric values in each cell give the corresponding r value.

Pearson correlation analysis of the 30 selected isolates showed only a few strong relationships between variables (Figure 6). Among enzymes, lipase activity correlated positively with urease (r = 0.47) and esterase (r = 0.34), while protease activity was negatively associated with coccobacilli occurrence (r = −0.69). The strongest links with plastic-degradation performance were observed for lipase and urease. Regarding to Clear Zone Method (CZM), lipase activity showed a moderate positive correlation with increase in optical-density–based growth (CZM(OD), r = 0.57) and a weaker correlation with CFU increase (CZM(CFU), r = 0.39). Urease activity also correlated with CZM(OD) (r = 0.36) and, to a lesser extent, with CZM(CFU) (r = 0.25). In contrast, catalase, esterase, and peroxidase showed little or no association with the three CZM metrics, and correlations with season, water type, or zone were generally weak. Overall, the heatmap indicates that lipase, and secondarily urease, are the phenotypic traits most closely aligned with plastic-degradation performance in this isolate set.

### 3.6 Molecular confirmation

Out of the eight isolates that demonstrated consistently positive plastic-degradation activity across all preliminary tests, four were selected for further analysis from diverse aquatic habitats: two from freshwater sources (Nile River and Nile Canal) and two from marine environments (Red Sea coast and Mediterranean Sea coast). These isolates were identified by the Identification Biotechnology Unit at the Reference Laboratory for Veterinary Quality Control on Poultry Production, Animal Health Research Institute, Dokki, Giza, Egypt. Molecular identification using PCR and sequencing revealed the presence of *Micrococcus luteus*, *Enterobacter cloacae*, *Corynebacterium aurimucosum*, and *Mesobacillus maritimus*.

**FIGURE 7:**
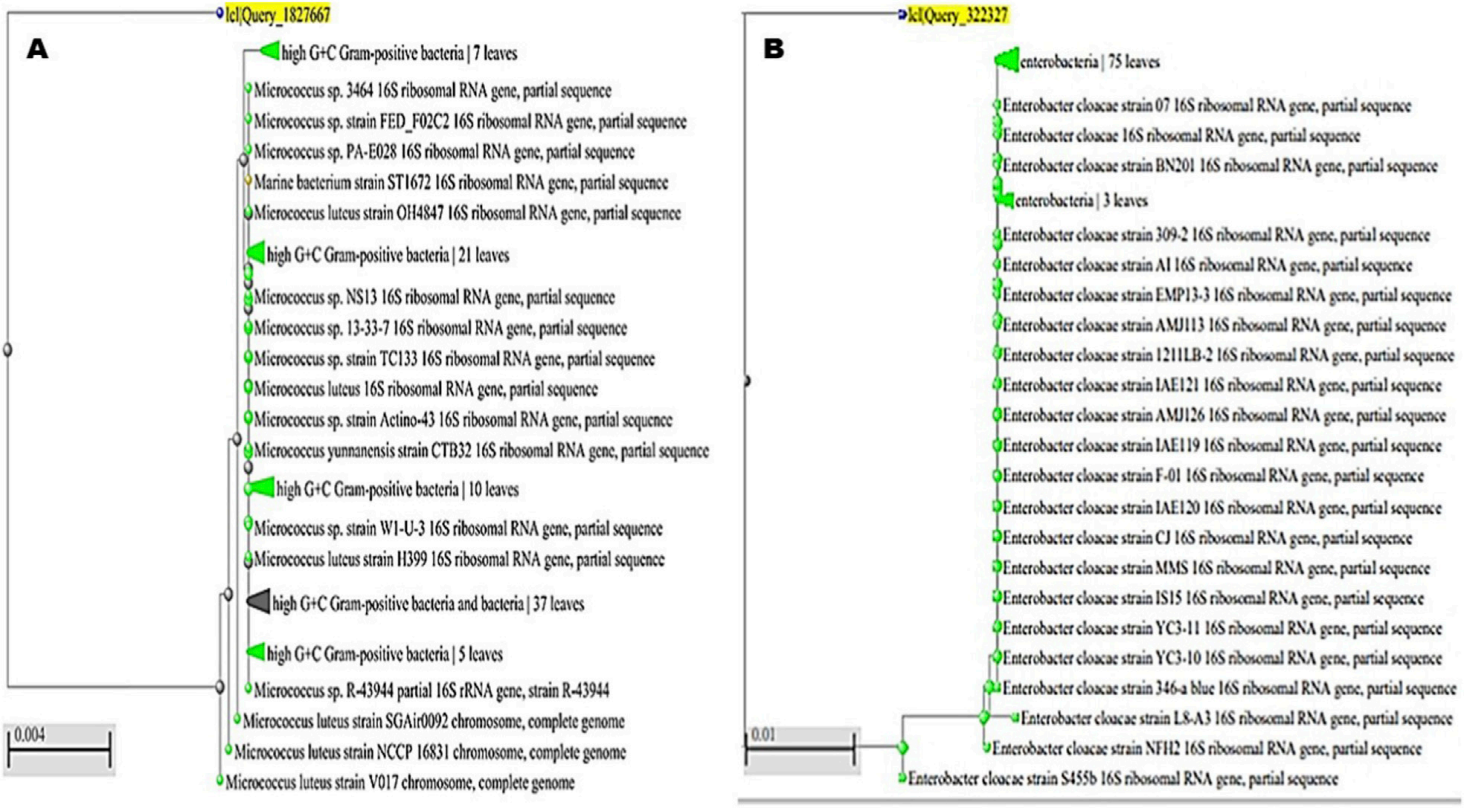
Phylogenetic analysis of bacterial isolates based on partial 16S rRNA gene sequences. **(A)** The first isolate clustered within the high G + C Gram-positive bacteria, showing close affiliation with *Micrococcus luteus* reference strains (e.g., OH4847, H399, NCCP 16831), confirming its identification as *Micrococcus luteus* (accession no. PQ772638). **(B)** The second isolate grouped within the Enterobacteriaceae, displaying strong relatedness to multiple E*nterobacter cloacae* reference strains (e.g., BN201, AMJ113, S455b), supporting its classification as *Enterobacter cloacae*.

**FIGURE 8:**
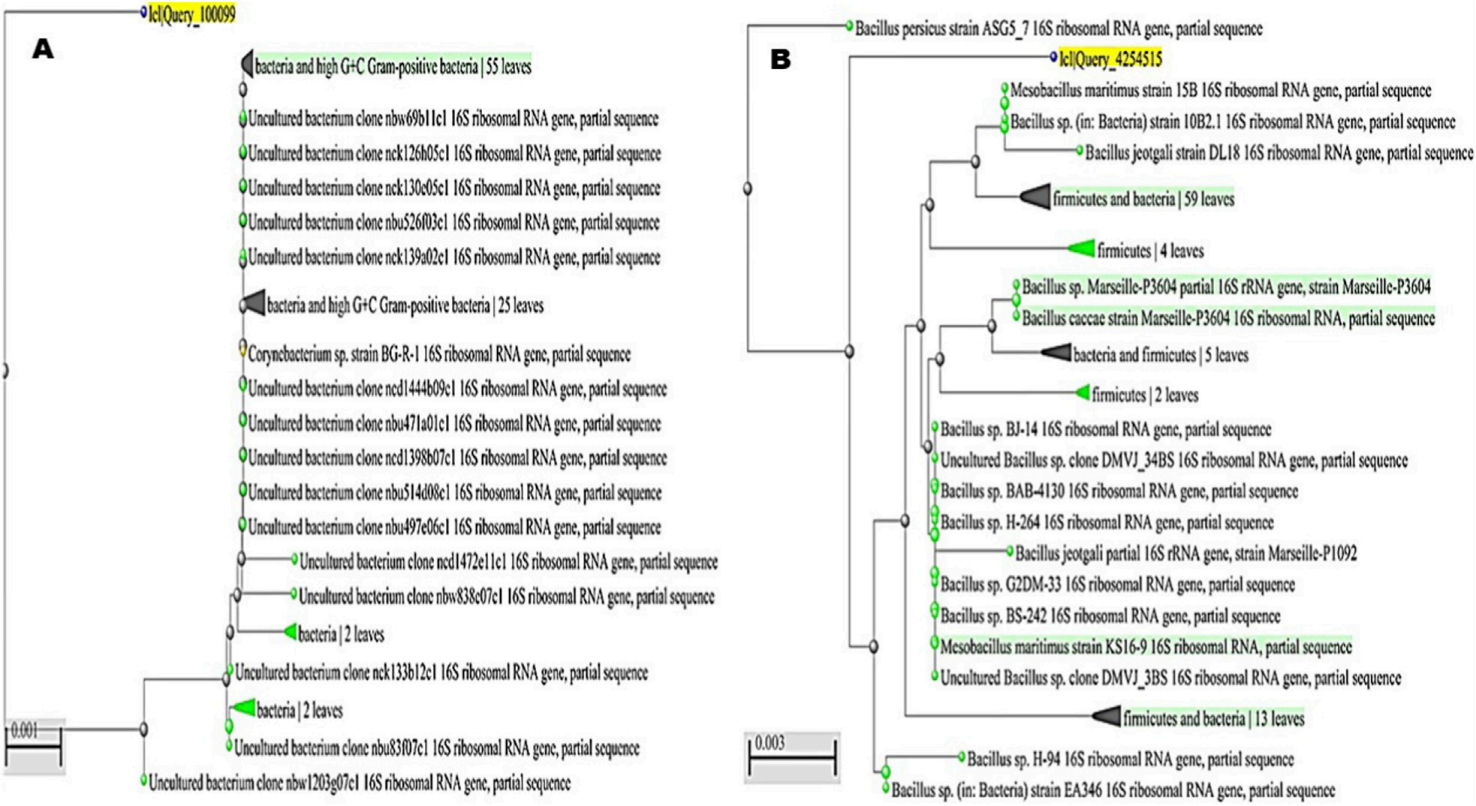
Phylogenetic placement of bacterial isolates based on partial 16S rRNA gene sequences. **(A)** The third isolate clustered within the high G + C Gram-positive bacteria, aligning closely with *Corynebacterium* sp. strain BG-R-1 and related uncultured bacterial clones, supporting its identification as *Corynebacterium aurimucosum* (accession no. PQ878353). **(B)** The fourth isolate grouped within the Firmicutes and was closely related to *Mesobacillus maritimus*, forming a distinct cluster with strains KS16-9 and 15B alongside related *Bacillus* species, confirming its classification as *Mesobacillus maritimus* (accession no. PV053511).

Partial 16S rRNA gene sequencing followed by phylogenetic analysis enabled the identification of the bacterial isolates recovered from the samples. The first isolate clustered firmly within the high G + C Gram-positive bacteria, showing close affiliation with *M. luteus* reference strains such as OH4847, H399, and NCCP 16831 (Figure 7A). This consistent grouping confirmed its classification as *M. luteus*, and the sequence was deposited under the accession number PQ772638. The second isolate grouped within the Enterobacteriaceae and showed a strong relationship to multiple *E. cloacae* reference strains, including BN201, AMJ113, and S455b (Figure 7B). Its placement within the well-supported *E. cloacae* complex verified its taxonomic identity, and the sequence was submitted to GenBank with the accession number PV111778. The third isolate was positioned within the high G + C Gram-positive bacteria, aligning closely with members of the genus *Corynebacterium*. It clustered alongside *Corynebacterium* sp. strain BG-R-1 and several uncultured bacterial clones, supporting its identification as *C. aurimucosum* (Figure 8A). The sequence was assigned the accession number PQ878353, establishing its molecular record for future comparative studies. The fourth isolate belonged to the phylum Firmicutes and was closely associated with the genus *Mesobacillus*. It formed a distinct cluster with *M. maritimus* strains KS16-9 and 15B, in addition to other related *Bacillus* species (Figure 8B). This phylogenetic relationship confirmed its designation as *M. maritimus*, and the sequence was deposited under the accession number PV053511.

### 3.7 Detection of environmental plastic pollution in the aquatic samples using HPLC

Plastic-derived contaminants such as terephthalic acid (TPA), bisphenol A (BPA), di(2-ethylhexyl) phthalate (DEHP), dibutyl phthalate (DBP), and dimethyl phthalate (DMP) showed varying distributions in freshwater and saltwater habitats. Wastewater treatment plant (WWTP) effluents were the most concentrated and consistent sources. Among the five WWTP samples, three contained the highest levels, with all of these contaminants detected. In saltwater, all compounds were found in only one coastal marina sample. The remaining marina and seashore samples showed either partial detection in three cases or no detectable contaminants in one case.

### 3.8 Detection of biodegradation of natural and synthetic plastics by selected isolates

#### 3.8.1 SEM analysis to confirm biodegradation

**FIGURE 9:**
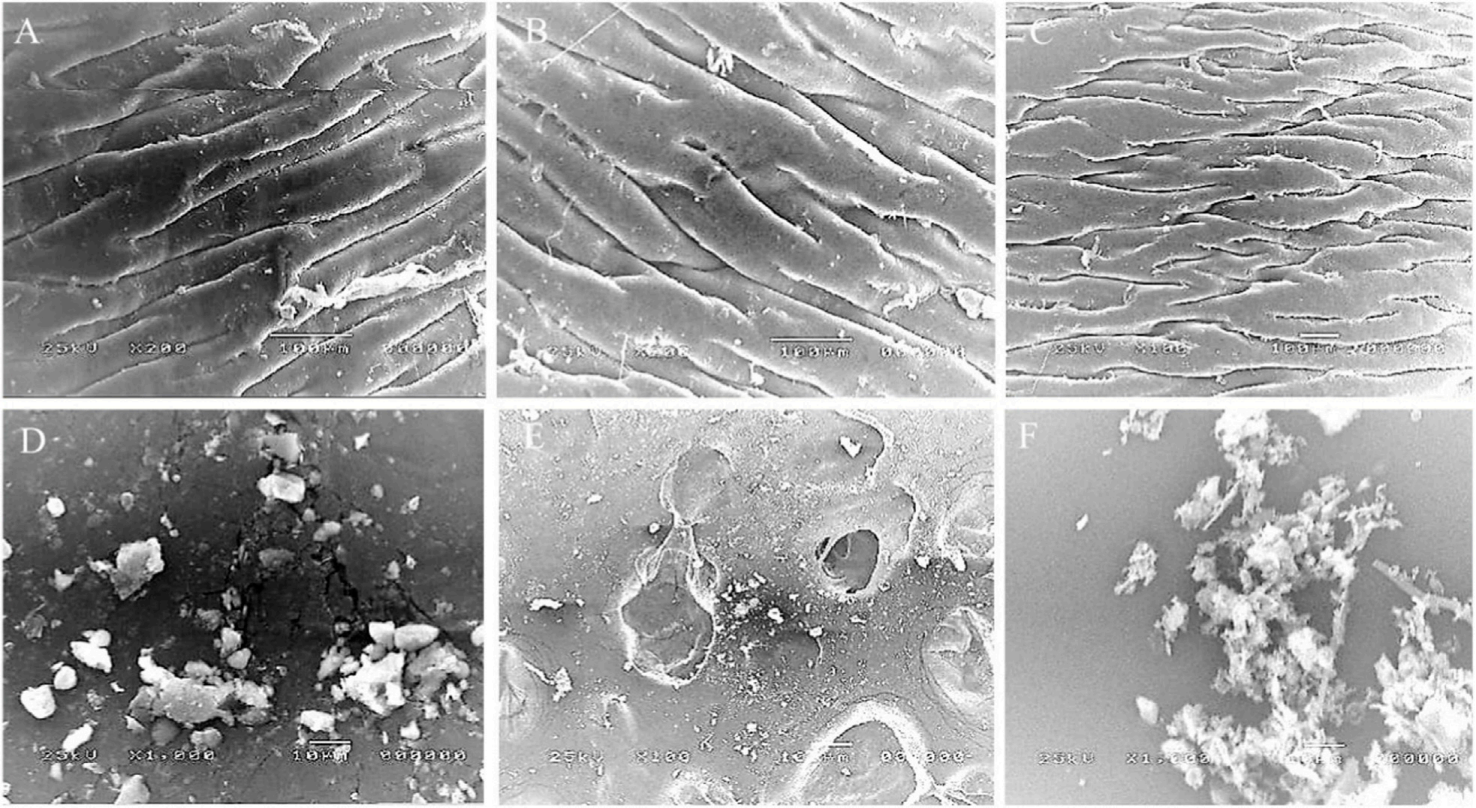
Scanning Electron Microscopy (SEM) analysis of plastic biodegradation. **(A–C)** Untreated plastics: **(A)** commercial sample **(B)** freshwater environmental sample, and **(C)** saltwater environmental sample, all showing relatively smooth and intact surfaces. **(D–F)** Plastics after microbial treatment: **(D)** commercial plastic exhibiting minor cracks and localized roughness indicating early degradation; **(E)** freshwater plastic showing pronounced erosion with pits and cavities; and **(F)** saltwater plastic displaying the most severe degradation with extensive fragmentation, rough textures, and amorphous debris accumulation. Together, these results highlight that naturally weathered plastics (freshwater and saltwater) undergo more extensive microbial degradation compared to commercial plastics under identical conditions.

Plastic biodegradability was assessed using a commercial sample along with the most highest plastic polluted environmental samples (one freshwater and one saltwater), analyzed by Scanning Electron Microscopy (SEM) and Proton Nuclear Magnetic Resonance (^1^H NMR) spectroscopy. Scanning Electron Microscopy (SEM) analysis revealed distinct morphological differences between commercial and natural plastic samples following biodegradation. The commercial plastic sample (Figure 9A) initially showed a relatively smooth and continuous surface morphology, characterized by parallel ridges and limited structural irregularities. After treatment with the mixture of selected bacterial isolates, only early signs of surface alteration were evident, including minor cracks and localized roughness, which indicated the onset of microbial degradation (Figure 9D). In contrast, the natural plastic polluted samples displayed more advanced structural damage. For the freshwater environmental plastic sample (Figure 9B), a uniform topography with no significant surface defects was observed prior to microbial treatment. However, the treated sample (Figure 9E) exhibited pronounced surface erosion characterized by pits and cavities, suggesting active microbial attack and polymer chain breakdown. Similarly, the untreated saltwater environmental plastic sample (Figure 9C) showed a relatively normal morphological appearance, whereas the treated sample (Figure 9F) demonstrated the most severe degradation, with extensive fragmentation, rough textures, and accumulation of amorphous debris, reflecting advanced biodegradation. Of note, treated environmental plastics showed significantly higher damage indices than treated commercial plastics p < 0.05. These results indicate that natural plastics, already subjected to environmental weathering, undergo more extensive microbial degradation compared to commercial plastics under similar conditions.

#### 3.8.2 ^1^H NMR spectral analysis of treated saltwater samples

**FIGURE 10:**
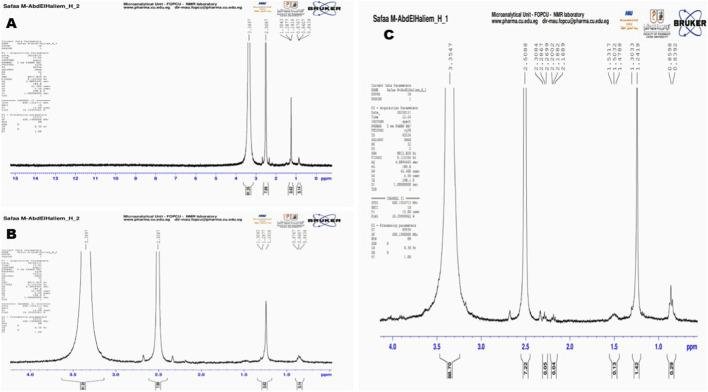
^1^H NMR spectral analysis of biodegraded plastic extracts. **(A)** Saltwater plastic-polluted sample showing characteristic resonances at δ 3.35 and δ 2.56 ppm, along with aliphatic signals (δ 1.30–0.84 ppm), indicating PET degradation products and phthalate additives. **(B)** Freshwater plastic-polluted sample with dominant ester-associated peaks at δ 3.35 and δ 2.56 ppm, plus aliphatic methyl/methylene resonances (δ 1.30–0.62 ppm), consistent with PET and phthalate breakdown. **(C)** Commercial plastic samples (LDPE, PHA, PET) displaying multiple resonances, including ester-linked fragments (δ 3.35, δ 2.50 ppm), aliphatic backbone signals (δ 1.53–1.24 ppm), and terminal methyl groups (δ 0.85 ppm), confirming microbial degradation of both synthetic and biodegradable polymers.

The ^1^H NMR spectrum of the saltwater plastic sample extract (recorded in DMSO-d_6_) displayed several well-defined proton resonances across the chemical shift range of 0.8–3.5 ppm (Figure 10A). Prominent signals were observed at δ 3.35 ppm and δ 2.56 ppm, which are characteristic of methylene protons adjacent to electronegative groups such as esters or aromatic moieties, consistent with degradation products of polyethylene terephthalate (PET) and related polyesters. Additional peaks at δ 1.30 ppm, δ 1.28 ppm, δ 1.24 ppm, and δ 0.86–0.87 ppm correspond to aliphatic–CH_2_– and–CH_3_ proton environments typically associated with phthalates and other plasticizer residues. Minor resonances near δ 0.84 ppm further support the presence of terminal methyl groups from branched alkyl chains, which are common in phthalate esters such as DEHP and DBP. The detection of these chemical shifts provides molecular-level evidence of polymer chain scission and additive release, thereby confirming that plastic biodegradation had occurred to the plastic pollutant within saltwater environments.

#### 3.8.3 ^1^H NMR -based evaluation of biodegraded freshwater sample

The ^1^H NMR spectrum of the freshwater plastic-polluted sample extract (recorded in DMSO-d_6_) showed distinct resonances in both aliphatic and oxygenated regions (Figure 10B). A dominant peak at δ 3.35 ppm indicated–CH_2_– groups adjacent to oxygen atoms in ester or ether linkages, consistent with PET and related polyester structures. A second major signal at δ 2.56 ppm was assigned to methylene protons adjacent to carbonyl groups (–CH_2_–C=O), supporting the presence of ester functionalities typical of PET and phthalate additives. Additional signals at δ 1.30–1.24 ppm and δ 0.86–0.84 ppm corresponded to terminal–CH_3_ and–CH_2_– groups from plasticizer side chains such as DEHP and DBP. Minor resonances at δ 0.62 ppm and δ 0.14 ppm suggested contributions from branched aliphatic chains commonly associated with freshwater-derived plastic debris.

#### 3.8.4 Chemical fingerprinting of treated commercial plastics samples

The ^1^H NMR spectrum of biodegraded commercial plastic samples, which included standardized forms of LDPE, PHA, and PET, revealed multiple distinct resonances corresponding to both polymer backbones and degradation products (Figure 10C). A strong resonance at δ 3.35 ppm was attributed to methylene protons adjacent to oxygen atoms (–CH_2_–O–), confirming the presence of ester linkages consistent with PET and PHA degradation products. A sharp signal at δ 2.50 ppm, along with multiple smaller peaks between δ 2.30–2.16 ppm, indicated methylene groups adjacent to carbonyl functionalities (–CH_2_–C=O), characteristic of both PET chain scission products and PHA degradation intermediates. Additional peaks observed at δ 1.53–1.24 ppm represent methylene and methine protons typical of LDPE aliphatic backbones as well as PHA side chains. Signals at δ 0.85–0.83 ppm correspond to terminal methyl groups (–CH_3_), further supporting contributions from LDPE degradation products. Minor resonances near δ 1.42 ppm and δ 0.29 ppm suggest the presence of branched aliphatic residues, likely reflecting oxidative breakdown of LDPE polymers. Collectively, the NMR data indicate that all three tested polymers underwent biodegradation under consortium activity, as evidenced by the detection of ester-linked fragments (PET and PHA) and aliphatic chain scission products (LDPE). The co-occurrence of PET-derived aromatic ester residues and LDPE aliphatic fragments confirms that the microbial consortium was able to target both synthetic and biodegradable polymers, leading to detectable molecular-level modifications.

## 4 Discussion

Microplastic pollution is now documented in marine, freshwater, soil, and even atmospheric compartments, and is recognized as a persistent global contaminant with ecological and human health impacts. Recent reviews highlight the rapid accumulation of microplastics, their role as carriers of toxic additives and pathogens, and their potential to disrupt food webs and biogeochemical cycles at large scales (Abahussain et al., 2025). These findings support the need for strategies that do not only focus on mechanical removal or physical containment but also explore biological solutions that target the polymer itself. Bioremediation has emerged as an essential strategy to address the escalating problem of plastic pollution, since microorganisms play a vital role in degrading and transforming synthetic polymers into less harmful products (Shah et al., 2008; Auta et al., 2017). Aquatic ecosystems, both freshwater and marine, harbor a remarkable diversity of microorganisms that play central roles in nutrient cycling, food web dynamics, and ecosystem health. This diversity encompasses bacteria, archaea, fungi, protists, and viruses, though bacteria and archaea dominate in terms of abundance and functional importance (Newton et al., 2011; Sunagawa et al., 2015). Many of these bacteria, including *Pseudomonas spp*. and *staphylococcus sp*, possess numerous biochemical enzymes (Bendary et al., 2016) that confer broad metabolic capabilities and enhance their potential contribution to bioremediation processes (Shah et al., 2008; Auta et al., 2017). In the present study, preliminary microbiological screening of water samples revealed substantial microbial diversity, with 178 samples (89%) showing positive growth and yielding 277 isolates. Comparisons between freshwater and saltwater samples confirmed that salinity acts as a key ecological filter influencing microbial community composition. Freshwater habitats are often dominated by Actinobacteria and other Gram-positive taxa (Newton et al., 2011). This pattern was consistent with our findings, where saltwater samples contained both Gram-positive and Gram-negative bacteria with diverse morphologies, while freshwater samples were dominated by Gram-positive cocci and rods, reflecting a simpler and more uniform community structure.

The biochemical screening revealed a broad range of enzymatic activities among the isolates. Catalase was the most prevalent, consistent with its well-known role in protecting microorganisms from oxidative stress by decomposing hydrogen peroxide (Chelikani et al., 2004). The widespread occurrence of catalase suggests that most isolates, irrespective of habitat, were adapted to environments where reactive oxygen species are commonly encountered. Protease activity was also frequent, underscoring the importance of extracellular proteolysis for nutrient acquisition and organic matter turnover in aquatic ecosystems (Rao et al., 1998). Lipase activity, detected in more than half of the isolates, is critical for the hydrolysis of lipids, particularly in nutrient-rich environments where lipids from organic detritus and pollutants are abundant (Jaeger and Eggert, 2002). In contrast, urease activity was the least represented, which may indicate that urea hydrolysis and its role in nitrogen cycling are specialized functions within these aquatic microbial communities (Mobley and Hausinger, 1989). Esterase and peroxidase displayed intermediate prevalence, reflecting their roles in ester degradation and oxidative stress defense, but their partial distribution also points to functional redundancy across the microbial consortia.

Seasonal analysis revealed notable patterns in microbial recovery and enzyme activities. The highest number of isolates were obtained during summer. This trend supports the notion that warmer temperatures enhance microbial growth and culturability, in line with studies reporting seasonal peaks in microbial abundance during warmer months (Crump et al., 2003). Enzyme activities also reflected seasonal influences: protease and to lesser extend catalase, remained consistently high across all seasons, indicating that oxidative stress defense and protein degradation are essential metabolic processes not strongly affected by environmental fluctuations. By contrast, lipase activity exhibited marked seasonality, with higher scores in autumn and winter, possibly linked to seasonal inputs of organic matter and temperature-driven lipid availability (Grossart et al., 2005). Worthy mention, these findings underscore the adaptability of aquatic microbes, with certain enzyme systems (e.g., catalase and protease) acting as core metabolic traits, while others (e.g., lipase and peroxidase) vary in response to environmental and seasonal factors. This functional diversity enhances microbial resilience and ecological contributions, particularly in biogeochemical cycling and pollutant degradation, including plastics and other organic contaminants.

The distribution of bacterial isolates across water types demonstrated that saltwater environments supported a greater proportion of isolates (79.5%) compared to freshwater (20.6%). This is consistent with evidence that marine systems often harbor higher microbial abundance and diversity due to their physicochemical stability and nutrient dynamics (Sunagawa et al., 2015; Giovannoni, 2017). The comparison of enzymatic activity revealed that most enzyme systems, including catalase, protease, esterase, urease, and peroxidase, maintained stable activity levels across both freshwater and saltwater environments (Chelikani et al., 2004; Rao et al., 1998). Interestingly, lipase activity exhibited clear sensitivity to water type, with maximum activity in saline environments. This aligns with previous studies reporting salt-tolerant lipase producers in marine bacteria, highlighting their ecological and biotechnological importance in lipid turnover and pollutant degradation in saline systems (Jaeger and Eggert, 2002; Joseph et al., 2008).

Source-specific comparisons revealed that marine habitats (Mediterranean and Red Sea) yielded the majority of isolates, far outnumbering freshwater sources (canals and Nile). This agrees with earlier findings that marine systems often act as reservoirs of metabolically versatile microorganisms (Grossart et al., 2005). Enzyme activity analysis showed that esterase remained stable across sources, indicating that these functions are constitutively maintained across aquatic habitats. In contrast, other enzyme varied markedly between sources, reflecting the influence of local environmental conditions such as nutrient loads, salinity, and organic matter composition. For example, elevated lipase activity in the Nile and Mediterranean samples may be linked to inputs of organic matter and pollutants in these regions, which select for lipid-degrading bacteria (Gao et al., 2024). Similarly, the high protease activity in Canal may reflect organic enrichment from anthropogenic or natural sources, driving protein degradation as a key microbial process.

Spatial zonation further highlighted habitat-specific trends. Bacterial recovery declined with increasing distance from the shoreline, suggesting higher microbial abundance and activity in nearshore zones where organic inputs and anthropogenic influences are more pronounced (Crump et al., 2003). Enzyme activities such as esterase remained uniform across zones (distance from the coastal area), pointing to their fundamental roles in microbial metabolism. However, other enzymes showed zone-specific variability. Protease and peroxidase activity peaked offshore (Zone D), which may reflect adaptation to organic substrates present in deeper or more saline waters. while catalase activity increased slightly in offshore samples, possibly linked to oxidative stress responses under different redox conditions. Such spatial variation in functional traits underscores the ecological adaptability of aquatic microbial communities and their ability to respond to environmental gradients.

Approximately one-third of the tested isolates, which harbored most of the key biochemical enzymes, demonstrated measurable plastic degradation in the preliminary screening assays. This proportion is consistent with reports from other aquatic and soil studies, where only a minority of environmental microbes show strong biodegradable capacity to attack synthetic polymers (Shah et al., 2008; Skariyachan et al., 2017). The relatively high percentage observed here (∼33%) suggests that plastic-polluted aquatic environments may serve as selective niches, enriching for microbial taxa with metabolic pathways capable of utilizing plastic substrates (Auta et al., 2017). These findings are ecologically significant, as they highlight the presence of naturally occurring microbial candidates capable of contributing to plastic biodegradation in aquatic systems. They also provide a foundation for future work involving enzyme characterization, whole-genome sequencing, and optimization of degradation conditions to evaluate their potential for biotechnological applications in plastic waste management.

The ecological relevance of the high-performing isolates is supported by their documented distribution and functional roles in natural aquatic systems. *Micrococcus luteus* is a common member of freshwater and marine biofilms and is known for robust oxidative and hydrolytic enzyme production, including catalases, esterases, and lipases that enable degradation of diverse organic substrates in low-nutrient environments (Zettler et al., 2013). *Enterobacter cloacae* is frequently detected in rivers, estuaries, wastewater-impacted sites, and coastal zones, where its metabolic versatility, rapid growth, and secretion of hydrolases support the breakdown of polymers and xenobiotics under fluctuating environmental conditions (Shah et al., 2008; Skariyachan et al., 2017). Parallel to these reports, *M. luteus*, *E. cloacae*, *C. aurimucosum*, and *M. maritimus* were molecularly confirmed as the most active plastic-biodegrading isolates in this study. Previous studies have highlighted the plastic-degrading potential of *M. luteus* and *E. cloacae*, particularly against polyethylene substrates, through biofilm formation and enzymatic action (Skariyachan et al., 2017; Das & Kumar, 2015).

*Corynebacterium aurimucosum*, while less intensively studied in plastic degradation, belongs to a genus widely reported from aquatic sediments and biofilms and is associated with esterase and oxidoreductase activities that contribute to polymer surface modification and early stages of biodegradation (Ojha et al., 2017). *Mesobacillus maritimus*, a Bacillus-related marine species, aligns with numerous Bacillus-type degraders known for secreting extracellular depolymerases, proteases, and lipases that remain active across broad temperature and salinity ranges (Urbanek et al., 2018). The natural occurrence of these organisms across diverse aquatic habitats, combined with their enzyme repertoires and biofilm-forming capacity, supports their relevance as biodegradation agents beyond laboratory conditions and highlights their potential role in *in-situ* bioremediation strategies. These observations were in line with our findings, as *C. aurimucosum* and *M. maritimus* have not been directly reported in plastic biodegradation, but their close taxonomic relatives, including *Corynebacterium* spp. and *Bacillus*/mesophilic *Bacillus*-like species, are known degraders of synthetic polymers (Ojha et al., 2017; Yoshida et al., 2016). Phylogenetic analysis based on 16S rRNA sequencing placed these isolates within well-defined clades: *M. luteus* grouped with established high G + C Gram-positive strains, *E. cloacae* within the Enterobacteriaceae, *C. aurimucosum* among Corynebacteria relatives, and *M. maritimus* within the Firmicutes, closely clustering with Bacillus-like strains. Finding these bacteria in different aquatic environments shows that many types of microbes may help break down plastics and that they are worth studying further to understand how they work.

HPLC analysis confirmed the presence of multiple plastic-derived contaminants, including terephthalic acid (TPA), bisphenol A (BPA), di(2-ethylhexyl) phthalate (DEHP), dibutyl phthalate (DBP), and dimethyl phthalate (DMP), across both freshwater and saltwater environments. Wastewater treatment plant (WWTP) effluents emerged as the most consistent and concentrated sources, with three of five samples containing all tested contaminants. This finding is in line with reports that WWTPs represent significant point sources for microplastics and plasticizers due to incomplete removal during treatment (Carr et al., 2016; Sun et al., 2019). In saltwater habitats, contamination was more heterogeneous: all compounds were detected in one marina, while other marina and seashore samples showed partial or no detection. This variability reflects localized inputs from human activity, boating, and coastal runoff, which are recognized contributors to microplastic and additive pollution in marine systems (Andrady, 2011; Gewert et al., 2015). The detection of plastic contaminants alongside the isolation of putative plastic-degrading bacteria underscores the ecological relevance of these microorganisms in polluted aquatic systems. It also suggests potential interactions between microbial communities and plastic-derived compounds, where bacteria capable of metabolizing xenobiotics may contribute to natural attenuation processes. Together, these findings strengthen the case for targeted exploration of environmental isolates as candidates for bioremediation of plastic pollution.

The combined use of SEM and ^1^H NMR provides molecular and morphological evidence supporting the biodegradation of both natural and synthetic plastics by selected bacterial isolates. This allowed for a comprehensive evaluation of polymer degradation, bridging the gap between surface-level structural alterations and chemical transformations at the molecular scale. Such a dual approach is increasingly recommended for assessing plastic biodegradation, as microscopy alone cannot fully capture underlying chemical modifications (Urbanek et al., 2018; Raddadi and Fava, 2019). The detection of degradation products from PET, PHA, and LDPE demonstrates that the microbial consortium deployed in this study possessed broad enzymatic capabilities. Previous research has highlighted that mixed microbial communities often outperform single strains in degrading complex polymers, owing to complementary enzymatic systems and metabolic cooperation (Yoshida et al., 2016; Skariyachan et al., 2017). In this context, the consortium comprising *M. luteus, E. cloacae, C. aurimucosum,* and *M. maritimus* proved particularly efficient. *Micrococcus luteus* has been reported to secrete oxidative enzymes that initiate polymer surface modifications, facilitating further enzymatic attack (Pathak and Navneet, 2017). *Enterobacter cloacae* is known for its metabolic versatility, including the degradation of phthalates and ester-linked compounds, which enhances PET and PHA breakdown (Rai et al., 2013). *Corynebacterium aurimucosum*, as a member of the coryneform group, contributes through hydrolytic activities on ester and amide linkages, while *M. maritimus*, a halotolerant species, likely supports degradation under saline conditions, improving efficiency in saltwater environments (Sivan, 2011; Sekiguchi et al., 2011). PET-derived ester fragments and LDPE aliphatic breakdown products identified by NMR in this study align with reported enzymatic activities of cutinases, lipases, and oxidoreductases known to act on polyesters and polyolefins (Wei and Zimmermann, 2017; Danso et al., 2019). Collectively, these findings suggest that the synergistic action of the four isolates not only accelerated the biodegradation of inherently biodegradable polymers such as PHA, but also extended activity to more recalcitrant plastics like LDPE, although to a lesser extent.

One of the key insights from this investigation is the observation that environmental plastic samples exhibited more pronounced degradation than standardized commercial plastics under identical microbial treatment conditions. This can be attributed to the pre-weathering effects that environmental plastics undergo *in situ*, including photodegradation, oxidative stress, and mechanical abrasion, which weaken polymer chains and create entry points for microbial attack (Gewert et al., 2015; Jacquin et al., 2019). Such preconditioning likely facilitated the enhanced microbial degradation observed in both freshwater and saltwater samples, whereas commercial plastics with relatively intact polymer matrices presented greater resistance. This finding underscores the importance of considering the environmental history of plastic debris when evaluating biodegradability.

The combined evidence of structural deterioration and molecular scission highlights the potential of microbial consortia in mitigating plastic pollution across diverse ecosystems. Importantly, the more advanced degradation observed in saltwater samples indicates that salinity and associated microbial adaptations may enhance biodegradation pathways. This finding supports previous reports that marine-derived microbes display unique metabolic adaptations that allow them to utilize hydrocarbons and synthetic polymers as carbon sources (Sekiguchi et al., 2011; Delacuvellerie et al., 2019). The ability to target both synthetic and biodegradable plastics further suggests that microbial treatments could be adapted for integrated bioremediation strategies addressing mixed plastic wastes.

Our dataset of enzyme activities and degradation metrics fits within this broader interdisciplinary trend. By combining traditional statistics with multivariate analyses and by aligning our work with recent advances in microbial plastic degradation and machine learning for environmental applications, the study contributes to ongoing efforts to link microbial function, environmental context, and quantitative metrics of plastic transformation. Referencing recent reviews on microplastic threats and microbial biodegradation (Ullah et al., 2024; Gao et al., 2024). Overall, our finding confirmed that aquatic bacterial isolates could be used to degrade both commercial and environmentally plastics under conditions that mimic natural waters. A key novel aspect is the identification of *M. maritimus* and related aquatic isolates as candidates for plastic degradation, which to our knowledge has not been reported before and expands the range of taxa considered in this context. The detected bacterial consortium that degrades several polymers in this study supports the idea that mixed communities can offer a more robust and flexible bioremediation strategy than single strains and highlights the potential of assembling targeted consortia from natural communities. These findings show that more kinds of bacteria are involved in plastic degradation than previously known and suggest that aquatic environments may contain many useful plastic degrading bacteria that have not yet been studied. More broadly, this study shows that aquatic bacteria can break down relevant plastic types under simple lab conditions that are close to real waters. This supports the use of biological tools as part of strategies to manage plastic pollution. We hope that these findings can be further developed by environmental and public health institutions as one of the approaches to mitigate plastic pollution.

## 5 Conclusion

This study emphasizes the critical roles of environmental preconditioning, microbial diversity, and polymer chemistry in determining plastic biodegradation outcomes. Harnessing indigenous aquatic microorganisms, particularly through consortium-based approaches, offers a promising pathway toward sustainable and eco-friendly plastic bioremediation. Advancing this line of research with mechanistic and quantitative insights will be essential for translating laboratory findings into effective field-scale applications. Ultimately, these findings strengthen the case for biological solutions to plastic persistence and highlight the need to integrate microbial bioremediation strategies into global efforts to mitigate plastic pollution.

### 5.1 Strengths and limitations

This study has several strengths. It uses a wide and systematic sampling design that covers freshwater and saltwater habitats, different seasons, and distance gradients from the shore, which makes the findings environmentally relevant. It combines culture-based isolation with detailed enzymatic profiling of key hydrolytic activities, giving a functional picture of the isolates rather than only taxonomic labels. The use of three complementary degradation assays together with 16S rRNA sequencing allows robust selection and confirmation of the most efficient plastic degraders. Structural and molecular tools such as SEM and NMR, in addition to HPLC detection of plastic-derived contaminants in real water samples, strongly support that true polymer degradation occurs and link it to actual pollution levels. The assembly and testing of a bacterial consortium on both commercial and environmentally plastics further strengthen the study by demonstrating a realistic bioremediation scenario with direct application potential.

While the current findings provide strong evidence of biodegradation, several limitations must be acknowledged. First, the SEM and NMR analyses confirm degradation signatures but do not quantify degradation rates or mass balance. Future studies should incorporate weight-loss assays, carbon dioxide evolution tests, or gel permeation chromatography to assess degradation kinetics. Second, this study used 16S rRNA gene sequencing with Sanger sequencing, which supports mainly genus-level identification and may not resolve closely related species. Precise species-level identification of all isolates was not the primary objective, as the main aim was to evaluate plastic biodegradation by aquatic bacterial isolates and consortia. This focus represents a limitation of the present work. Future studies should apply whole-genome sequencing to the four key isolates to refine their taxonomic assignment, achieve species-level resolution, and better describe their genetic potential. Future studies should also integrate genomic data with enzymatic activity profiles and annotate genes involved in polymer degradation and related pathways to link genotype to the observed functions. For the consortia, future studies should use metagenomic sequencing at multiple time points during incubation on plastic to track community composition, relative abundances, and functional genes associated with plastic degradation over time.

## Data Availability

The data presented in the study are deposited in the https://www.ncbi.nlm.nih.gov/nuccore/PV111778, https://www.ncbi.nlm.nih.gov/nuccore/PQ878353, https://www.ncbi.nlm.nih.gov/nuccore/PV053511 repository, accession number PV111778, PQ878353, and PV053511.
