# Supplementary material for: Toward sustainable plastic bioremediation using bacterial consortia from aquatic environments
Source: Front Bioeng Biotechnol. 2026 Jan 5;13:1709072. doi: 10.3389/fbioe.2025.1709072 (PMC12812918; doi:10.3389/fbioe.2025.1709072)
Supplement: Supplementary file 1 [file Table1.docx]

# **Table S1. Summary of controls used in each assay and their experimental purpose**

| **Assay / section** | **Test condition** | **Controls used** | **Purpose of control** |
| --- | --- | --- | --- |
| Biochemical enzyme tests (catalase, peroxidase, urease, lipase, protease, esterase) | Test isolate on specific diagnostic medium or reagent | Reference positive strain; reference negative strain | Validate that the assay and reagents work as expected and that scoring of test isolates is reliable |
| Preliminary plastic degradation – Clear Zone Method (CZM) | BMM agar + plastic (0.1–0.5% w/v) + test isolate | BMM + plastic, no cells (negative control) | Detect abiotic clearing or contamination; define background |
|  |  | BMM + 0.2% glucose + test isolate (positive growth control) | Confirm viability of the inoculum and that the medium supports growth |
|  |  | BMM without added carbon + test isolate (inoculum control) | Check survival of the inoculum in carbon-free conditions and distinguish true plastic utilisation from carry-over carbon |
|  |  | BMM + emulsifier (0.05% Tween-80), no plastic + test isolate (matrix control) | Control for any effect of the emulsifier or medium components on halo formation |
| Preliminary plastic degradation – OD600 growth in liquid BMM | Liquid BMM + plastic (0.5–1% w/v or one disc) + test isolate | Same four controls as above (negative, positive growth, inoculum and matrix) | Separate true plastic-supported growth from background growth, inoculum carry-over or emulsifier effects |
| Preliminary plastic degradation – CFU counts in liquid BMM | Liquid BMM + plastic + test isolate, plated at intervals | Same four controls as above (negative, positive growth, inoculum and matrix) | Confirm that increases in CFU reflect growth on plastic rather than residual carbon or matrix components |
| Consortium plastic degradation for SEM/NMR | Carbon-limited Bushnell–Haas or artificial seawater + plastic coupon + bacterial consortium | Medium + plastic coupon, no consortium (uninoculated plastic control) | Monitor abiotic changes to the plastic surface or leaching in the absence of microbes |
|  |  | Medium + consortium, no plastic (consortium-only control) | Detect growth of the consortium on residual medium components and separate this from plastic-derived growth |
|  |  | Sterilised plastic coupon only (handling control) | Verify that sterilisation and handling do not induce structural artefacts in SEM or NMR analyses |
| HPLC detection of environmental plastic-derived compounds | Environmental water extract injected on HPLC with external standards | Calibration standards (TPA, BPA, DEHP, DBP, DMP) | Quantitative identification of plastic-related compounds |

**Table S2 : Phenotypic and biochemical characteristics of 30 bacterial isolates with high enzymatic activities selected** **for preliminary screening of plastic degradation**

| Sample | Source | Zone | Season | G-ve | G+ve | Cocci | Cocco-  bacilli | Rods | Catalase | Lipase | Urease | Protease | Esterase | Peroxidase | CZM  (HF) | CZM  (OD) | CZM  (CFU) |
| --- | --- | --- | --- | --- | --- | --- | --- | --- | --- | --- | --- | --- | --- | --- | --- | --- | --- |
| R8 | Cyrene Island | Zone C | Autumn | - | + | + | - | - | + | - | - | + | + | + | + | - | + |
| R13 | Cyrene Island | zone A | Autumn | - | + | - | - | + | + | + | - | + | + | - | + | + | + |
| R21 | Dahab | zone A | Summer | - | + | + | - | - | + | + | + | + | + | + | - | - | - |
| R22 | Dahab | zone B | Autumn | - | + | + | - | - | + | + | + | + | + | + | + | + | + |
| R40 | Al Ain Al Sokhna | zone A | Winter | - | + | - | + | - | + | + | + | - | + | + | - | - | - |
| R45 | Al Ain Al Sokhna | zone D | Autumn | - | + | - | - | + | + | + | + | + | + | - | + | + | + |
| R73 | South Sainai | zone A | Autumn | + | - | - | - | + | + | + | - | + | + | + | + | + | + |
| R82 | South Sainai | Zone B | Winter | - | + | - | - | + | + | + | + | + | + | - | - | - | - |
| R94 | Hurghada | zone D | Spring | - | + | - | - | + | + | + | - | + | + | + | + | + | + |
| R103 | Hurghada | zone B | Spring | - | + | - | - | + | + | + | - | + | + | + | + | + | + |
| M3 | Port-Said | zone A | Spring | - | + | - | - | + | + | + | + | + | + | + | - | - | - |
| M7 | Port-Said | zoneC | Summer | - | + | + | - | - | + | + | + | + | + | + | + | + | + |
| M37 | Alexandria | zone B | Winter | - | + | + | - | - | + | + | + | + | + | - | + | + | + |
| M42 | Alexandria | zoneC | Summer | + | - | - | - | + | + | + | + | + | + | + | - | - | - |
| M60 | Kafr Elsheikh | zone B | Winter | - | + | - | - | + | + | + | - | + | + | + | - | - | - |
| M65 | Kafr Elsheikh | Zone A | Summer | - | + | - | - | + | + | + | - | + | + | + | - | - | - |
| M83 | Marina | zone B | Winter | - | + | - | - | + | + | + | + | + | + | - | + | + | + |
| M92 | Marina | zone B | Summer | - | + | - | - | + | + | + | - | + | + | + | + | + | + |
| M94 | Damietta | zone D | Summer | + | - | + | - | - | + | + | + | + | + | - | - | - | - |
| M113 | Damietta | zone B | Summer | - | + | - | - | + | + | - | - | + | + | + | + | + | - |
| C4 | Dessok Canal | zone A | Summer | - | + | - | - | + | + | - | - | + | + | + | + | - | + |
| C10 | Dessok Canal | zone B | Summer | - | + | + | - | - | + | + | + | + | + | + | + | + | + |
| C14 | Ibrahimiya Canal | zone B | Autumn | - | + | - | - | + | + | - | - | + | + | + | - | - | - |
| C17 | Ibrahimiya Canal | zone A | Spring | - | + | + | - | - | + | + | + | - | + | + | + | + | + |
| C28 | Mahmoudiyah Canal | zone A | Spring | + | - | - | - | + | + | - | - | + | + | + | - | - | - |
| C36 | Mahmoudiyah Canal | zone A | Autumn | + | - | - | - | + | + | + | + | + | - | + | + | + | + |
| C46 | Ismailia Canal | Zone A | Autumn | - | + | - | - | + | + | + | + | + | + | - | + | + | + |
| C50 | Ismailia Canal | Zone A | Summer | - | + | - | - | + | + | + | + | + | + | + | - | - | - |
| N2 | Nile river | zone A | Summer | - | + | - | - | + | + | - | - | + | - | + | + | + | + |
| N5 | Nile river | Zone A | Summer | + | - | - | - | + | + | - | + | + | - | - | + | + | + |

Red Sea isolates (R), Mediterranean Sea isolates (M), Nile river isolates (N), canal isolates (C), Gram negative (G−ve), Gram positive (G+ve), halo formation in Clear Zone Method (CZM HF), optical density at 600 nm in Clear Zone Method (CZM OD), colony-forming units in Clear Zone Method (CZM CFU), positive results (+), negative results
