# Supplementary figures and images for: Toward sustainable plastic bioremediation using bacterial consortia from aquatic environments
Source: Front Bioeng Biotechnol. 2026 Jan 5;13:1709072. doi: 10.3389/fbioe.2025.1709072 (PMC12812918; doi:10.3389/fbioe.2025.1709072)

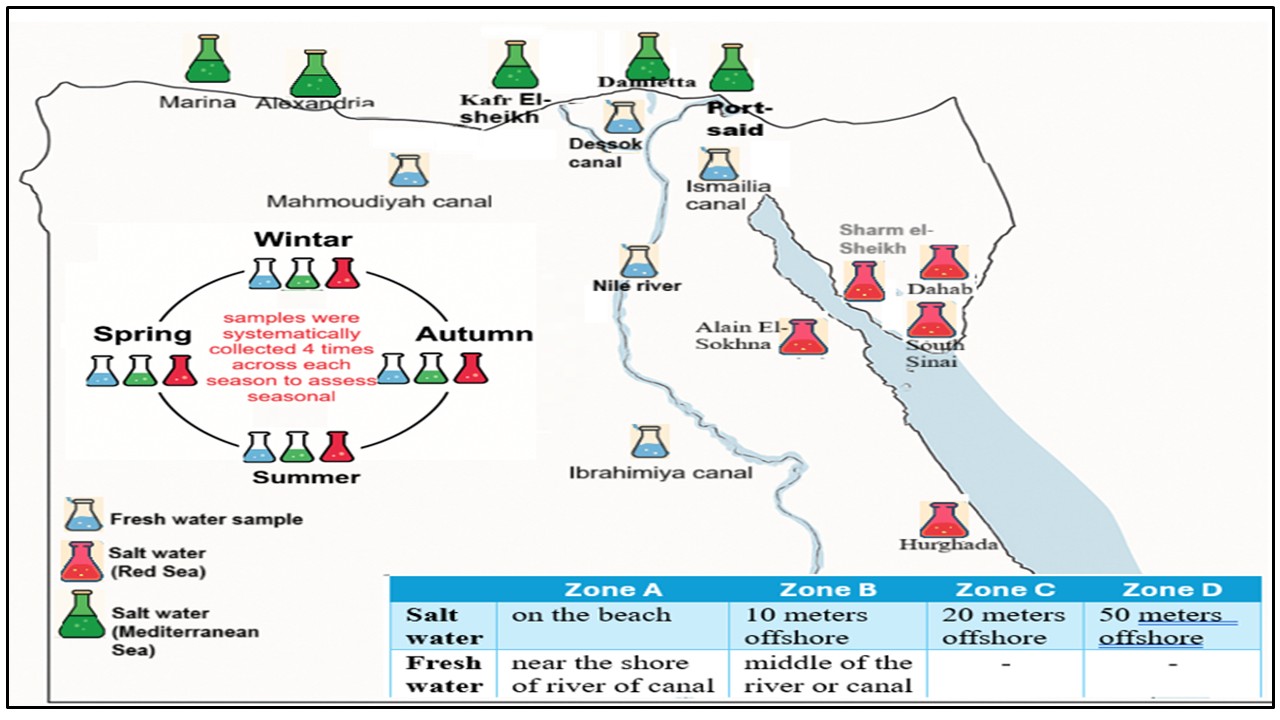

Supplement: Supplementary file 2 [file Image1.jpeg]
